# From Topological Analyses to Functional Modeling: The Case of Hippocampus

**DOI:** 10.3389/fncom.2020.593166

**Published:** 2021-01-11

**Authors:** Yuri Dabaghian

**Affiliations:** Department of Neurology, The University of Texas McGovern Medical School, Houston, TX, United States

**Keywords:** spatial learning, hippocampus, topological methods, place cells, theoretical model

## Abstract

Topological data analyses are widely used for describing and conceptualizing large volumes of neurobiological data, e.g., for quantifying spiking outputs of large neuronal ensembles and thus understanding the functions of the corresponding networks. Below we discuss an approach in which convergent topological analyses produce insights into how information may be processed in mammalian hippocampus—a brain part that plays a key role in learning and memory. The resulting functional model provides a unifying framework for integrating spiking data at different timescales and following the course of spatial learning at different levels of spatiotemporal granularity. This approach allows accounting for contributions from various physiological phenomena into spatial cognition—the neuronal spiking statistics, the effects of spiking synchronization by different brain waves, the roles played by synaptic efficacies and so forth. In particular, it is possible to demonstrate that networks with plastic and transient synaptic architectures can encode stable cognitive maps, revealing the characteristic timescales of memory processing.

## 1. Introduction

Spatial cognition in mammals is based on an internal representation of their environments—a cognitive map—used for spatial planning, navigating paths, finding shortcuts, remembering the location of the home nest, food sources and so forth. A central role in producing these maps is played by the hippocampal neurons famous for their spatially tuned spiking activity. In rats, these neurons, known as “place cells,” fire in specific domains of the navigated environment—their respective “place fields” (O'Keefe and Nadel, 1978; Moser et al., 2008). Thus, the spatial layout of the place fields in a given environment E—a place field map $M_{E}$—defines the temporal order in which place cells fire during animal's moves (Schmidt and Redish, 2013; Agarwal et al., 2014), and can therefore be viewed as a geometric “proxy” of the animal's cognitive map.

Experiments in “morphing” 2*D* environments demonstrate that place field maps are flexible: if the environment is deformed, then the place fields may change their shapes, sizes and locations, while preserving mutual overlaps, adjacencies, containments, etc. (Gothard et al., 1996; Leutgeb et al., 2005; Touretzky et al., 2005; Wills et al., 2005; Dabaghian et al., 2014; Bellmund et al., 2020; Place and Nitz, 2020). Hence the sequences in which the place cells fire during animal's navigation remain largely invariant within a certain range of geometric transformations, which suggests that the hippocampus provides a qualitative, topological representation of space—more akin to a subway map than a than to a topographical city street map (Alvernhe et al., 2012; Dabaghian et al., 2014; Wu and Foster, 2014).

The mechanisms that produce cognitive maps and the computational principles by which the brain converts patterns of neuronal firing into global representations of external space remain vague. Broadly, it is believed that the information provided by the individual place cells is somehow combined into single coherent whole. However, this “fusion” should not be viewed as a naïve aggregation of the smaller “pieces,” because the signals provided by the individual neurons have no intrinsic spatial attributes; rather, spatial properties are *emergent*, i.e., appearing at a neuronal ensemble level (Wilson and McNaughton, 1993; Pouget et al., 2000; Postle, 2006).

A computational framework developed in Dabaghian et al. (2012), Arai et al. (2014), Hoffman et al. (2016), Basso et al. (2016), Babichev et al. (2016a,b), Babichev and Dabaghian (2018), Dabaghian (2019), and Dabaghian (2016) helps to understand these phenomena by integrating the activity of the individual neurons into a large-scale map of the environment and to study the dynamics of its appearance, using algebraic topology techniques. Below we review some basic ideas and key concepts used in this framework, and discuss how they may apply to hippocampal physiology and cognitive realm. We then outline several examples that demonstrate how various characteristics of individual cells and synapses can be incorporated into the model and what effect these “microscopic” parameters produce at a “macroscale,” i.e., in the map that they jointly encode.

## 2. Topological Model

### 2.1. Alexandrov-Čech's Theorem

The topological nature of the cognitive map suggests that the information transmitted via place cell spiking should be amenable to topological analyses. For example, a place field map can be viewed as a cover of the environment E by the place fields υ_*i*_, $E=\cup_{i}\upsilon_{i}$, which, according to the Alexandrov-Čech's theorem (Alexandroff, 1928; Čech, 1932), encodes the topological shape of E. To evaluate its specific characteristics, one constructs the nerve of the cover—an abstract simplicial complex N whose simplexes, ν_*i*_0_, *i*_1_, …, *i*_*k*__ = [υ_*i*_0__, υ_*i*_1__, …υ_*i*_*k*__], correspond to non-empty overlaps between the place fields, υ_*i*_0__ ∩ υ_*i*_1__ ∩ … ∩ υ_*i*_*k*__ ≠ ∅. If these overlaps are contractible, then N has the same topological shape as E, i.e., the same number of components, holes, tunnels, etc. (Hatcher, 2002). An implication of this construction is that if the place fields cover the environment sufficiently densely, then their overlaps encode the topology of E, which provides a link between the place cells' spiking pattern and the topology of the represented space (De Silva and Ghrist, 2007; Curto and Itskov, 2008; Chen et al., 2012; Dabaghian et al., 2012; Kang et al., 2020).

### 2.2. Temporal Coactivity Complex

From the physiological perspective, the arguments based on the analyses of place fields provide only an indirect description of the information processing in the brain. In reality, the hippocampus and the downstream brain regions do not have access to the shapes and the locations of the place fields, which are but artificial constructs used by experimentalists to visualize their data. In the brain, the information is transmitted via neuronal spiking activity: if the animal enters a location where several place fields overlap, then there is a *probability* that the corresponding place cells will produce spike trains that overlap *temporally* (Curto and Itskov, 2008; Dabaghian et al., 2012). Such coactivities may be interpreted *intrinsically* by the downstream brain areas, and integrated into a global map of the ambient space. Thus, a proper description of place cell (co)activity requires a *temporal* analog of the nerve complex, built using temporal relationships between spike trains—which is, in fact, straightforward. Indeed, since the place field overlaps represent place cells' coactivities, one can construct a “coactivity complex” T whose simplexes correspond to combinations of active place cells, σ = [*c*_*i*_0__, *c*_*i*_1__, …, *c*_*i*_*k*__]. It was shown in De Silva and Ghrist (2007), Curto and Itskov (2008), and Dabaghian et al. (2012) that if such a complex is sufficiently complete (i.e., if it incorporates a sufficient number of the coactivity events) then its structure is similar to the structure of the spatially-derived nerve complex N, e.g., T correctly captures the topology of the physical environment. Note however, that structural similarity between N and T (representability of T) is a non-trivial point with profound mathematical implications (Tancer, 2013).

### 2.3. Simplicial Schemas of Cognitive Maps

Both complexes N and T provide a contextual framework for representing spatial information encoded by the place cells (Babichev et al., 2016b). For example, a sequence of place fields traversed during the rat's moves over a particular trajectory γ and the place cell combinations ignited along this trajectory can be represented, respectively, by a “nerve path” $\Gamma_{N}=\left\{\nu_{1},\nu_{2},\ldots ,\nu_{k}\right\}$—a chain of nerve-simplexes in N, or by a “coactivity path” $\Gamma_{T}=\left\{\sigma_{1},\sigma_{2},\ldots ,\sigma_{k}\right\}$—a chain of the coactivity-simplexes in T (see also Babichev and Dabaghian, 2018). These simplicial paths qualitatively represent the shape of the physical trajectories: a closed simplicial path represents a closed physical route; a non-contractible simplicial path corresponds to a class of the physical paths that enclose unreachable or yet unexplored parts of the environment; two topologically equivalent simplicial paths Γ_1_ ~ Γ_2_ represent physical paths γ_1_ and γ_2_ that can be deformed into one another and so forth (Brown et al., 1998; Jensen and Lisman, 2000; Guger et al., 2011; Dabaghian, 2016). By the Alexandrov-Čech's theorem, the net pool of the simplicial paths can thus be used to describe the topological connectivity of the environment E via homological characteristics of N and T.

### 2.4. The Large-Scale Topology of the Cognitive Map

C(E), as represented by a coactivity complex, can be described at different levels. A particularly concise description of a topological shape is given in terms of its topological loops (surfaces identified up to topological equivalence) in different dimensions, i.e., by its Betti numbers *b*_*n*_, *n* = 0, 1, … (Alexandrov, 1965; Hatcher, 2002). For example, the number of inequivalent topological loops that can be contracted to a zero-dimensional (0*D*) vertex, $b_{0}\left(T\right)$, corresponds to the number of the connected components in T; the number of loops that contract to a one-dimensional (1*D*) chain of links, $b_{1}\left(T\right)$, defines the number of holes and so forth (Alexandrov, 1965; Hatcher, 2002). The full list of the Betti numbers of a space or a complex *X* is known as its topological barcode, $𝔟\left(T\right)=\left(b_{0}\left(T\right),b_{1}\left(T\right),b_{2}\left(T\right),\ldots \right)$, which captures the topological identity of T (Zomorodian, 2005; Zomorodian and Carlsson, 2005; Ghrist, 2008; Carlsson, 2009, 2013; Edelsbrunner and Harer, 2010). For example, the barcode 𝔟 = (1, 1, 0, …) corresponds to a topological annulus, the barcode 𝔟 = (1, 0, 1, 0, …)—to a two-dimensional (2*D*) sphere *S*^2^, the barcode 𝔟 = (1, 2, 1, 0, …)—to a torus *T*^2^ and so forth (Edelsbrunner and Harer, 2010). Thus, by comparing the barcode of the coactivity complex 𝔟(T) to the barcode of the environment 𝔟(E) one can establish whether their topological shapes may match, i.e., whether the coactivity complex provides a faithful representation of the environment at a given moment *t*.

### 2.5. A Model of Spatial Learning

A key difference between the complexes N and T is that the topological shape of N is fully defined by the structure of the place field map, whereas the shape of T unfolds in time at the rate with which the spike trains are produced. At every given moment of time, the coactivity complex T represents connections between the place fields that the animal had time to “probe”: as the animal begins to explore a new environment, T is small, fragmented and may contain gaps that represent lacunae in the animal's internal map of the navigated space, rather than physical obstacles or inaccessible spatial domains. As the animal continues to navigate, more combinations of coactive place cells contribute connectivity information, the coactivity complex grows, $T\left(t\right)\subseteq T\left(t^{\prime }\right)$, *t* < *t*′, and acquires more details, converging to a stable shape that captures the physical structure of the surroundings.

**Figure 1:**
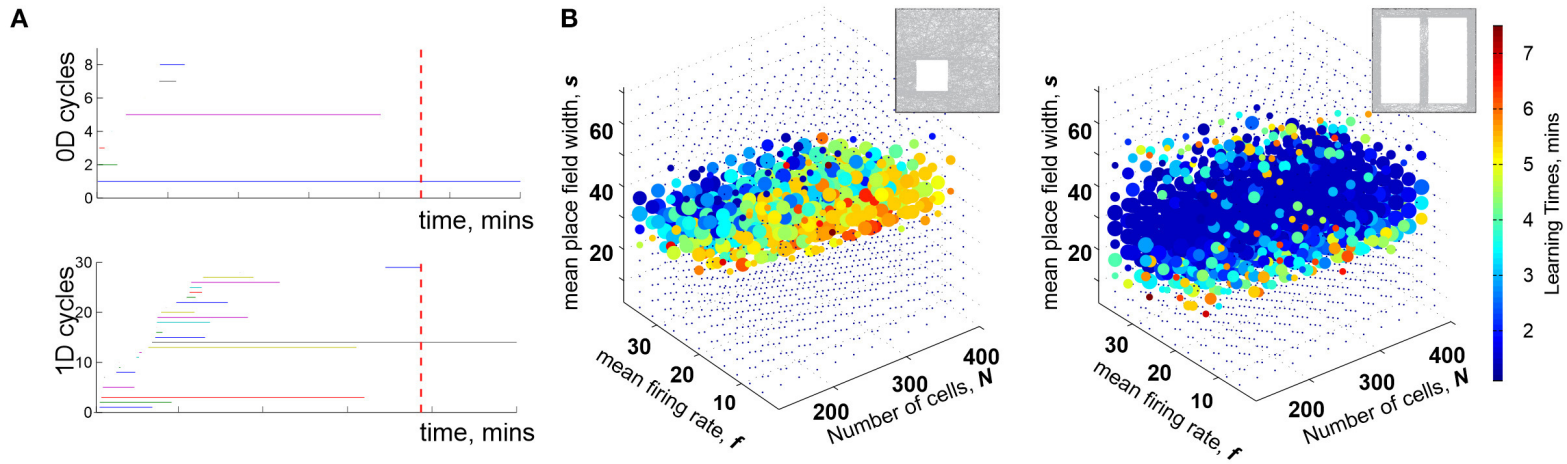
Topological description of spatial learning. **(A)** As the rat begins to explore an environment, the simplicial complex T(t) contains many 0*D* cycles that mark contractible spatial domains represented by small groups of cofiring place cells. Additionally, there exists many 1*D* cycles that represent transient holes in T(t). As exploration continues, spurious cycles disappear, leaving behind only a few persisting ones, which express stable topological information. **(B)** Each point in the parameters space with coordinates (*s, f, N*) represents a particular place cell ensemble. The colors of the dots represent the mean learning time *T*_*min*_; the larger the dot, the higher the rate of capturing the correct topological information for the corresponding (*s, f, N*). The ensembles that can produce a correct map occupy a particular domain of the parametric space—the *Learning Region*, L, where learning is fastest and most accurate; near the boundary, map forms over times and exhibits higher error rates. Outside of L learning fails. Importantly, the parameter values that correspond to L happen to parallel experimentally derived values, which indicates a biological relevance of the model. The smaller L on the left panel corresponds to a 2*D* arena with a hole, about 1.5 × 1.5 m in size, and the larger L on the right corresponds to a quasi-linear environment (top right corner of each panel). The more complex the environment, the more tuned the neural ensembles have to be to learn the space.

Mathematically, T can thus be viewed as a *filtered* complex, with the filtration defined by the times of the simplexes' first appearance, *t*_σ_ (Dabaghian et al., 2012). Methods of the Persistent Homology theory allow describing the dynamics of the topological loops in T, e.g., evaluating the minimal time *T*_min_ after which the topological structure of T matches the topology of the environment, $b_{n}\left(T\right)=b_{n}\left(E\right)$ (De Silva and Ghrist, 2007; Curto and Itskov, 2008; Dabaghian et al., 2012). Biologically, this value provides a low-bound theoretical estimate for the time required to learn a novel topological map from place cell outputs (Figure 1A) (Dabaghian et al., 2012; Arai et al., 2014; Babichev et al., 2016a,b, 2018; Basso et al., 2016; Hoffman et al., 2016; Dabaghian, 2019).

### 2.6. Facing the Biological Realm

The physiological viability of these algebraic-topological constructions depends on the parameters of neuronal firing activity: just as there must be a sufficient number of place fields covering a space in order to produce a topologically correct nerve complex N, certain conditions must be met by the place cell spiking profiles in order to produce an operational coactivity complex T. For example, there should be enough cofiring of place cells with sufficient spatial specificity of spiking; the encoded relationships should not be washed out by noise; the model should make realistic predictions, e.g., produce viable learning periods in different environments, etc. Given that biological systems are highly variable (Fenton and Muller, 1998), these criteria may or may not be met by the physiological place cell ensembles, or vice versa, the model may single out a certain “operational” scope of parameters that may not match the biological range. In the following, we discuss this and other correspondences between the topological model and hippocampal physiology. We demonstrate that, first, the model can incorporate a vast scope of physiologically relevant characteristics of spike times, spiking statistics, their modulations by the “brain waves,” efficacies of synaptic connections, architectures of the neuronal networks, etc., all of which correlate with dynamics of spatial learning. Second, the model allows converting this information *consistently* into coherent, biologically viable descriptions of a wide scope of neurophysiological phenomena. It becomes possible to systematically deduce functional properties of the system following not just the only empirical observations or experimental line of reasoning that currently dominate neurophysiological literature, but also the models' own, intrinsic logic.

### 2.7. Parameterization

To cope with the complexity of the cognitive map's construction, the model is built hierarchically: its main components implement most prominent physiological phenomena, and more subtle effects are incorporated as modifications of the skeletal structures. In the following, we will proceed in steps, by selecting a specific phenomenon, embedding it into the model using a minimal set of tools, outlining the results and discussing biological implications.

To simplify the approach, we will describe neuronal spiking in terms of Poisson firing rates, which, in case of the place cells, can be approximated by Gaussian functions of rat's coordinates with the amplitude *f*_*i*_ (the *i*th place cell's maximal firing rate), and the width *s*_*i*_ (the size of the corresponding place field) (Barbieri et al., 2004; Dabaghian et al., 2012). For an ensemble of *N* place cells, the *N* values *s*_*i*_ and *f*_*i*_ can be viewed as instantiations of two random variables drawn from their respective distributions with certain modes (*s* and *f* correspondingly) and standard deviations, σ_*s*_ and σ_*f*_. To avoid overly broad or overly narrow distributions we impose additional conditions σ_*s*_ = *bs* and σ_*f*_ = *af* with the coefficients *a* and *b* selected so match the experimental statistics (Brunel et al., 2004; Barbour et al., 2007; Buzsáki and Mizuseki, 2014). As a result, each specific place cell ensemble can be indexed by a triplet of parameters, (*s, f, N*).

Second block of parameters characterizes animal's behavior, e.g., speeds and trajectory shapes, which are computationally intractable. We assume a practical approach to this problem and simulate non-preferential exploratory spatial behavior, with no artificial moving patterns or favoring of one segment of the environment over another, with typical experimentally observed speed ranges. Such approach allows reproducing a natural flow of spiking data and estimating how long it takes to integrate it into a topological map. The statistical alternatives for the model are produced by randomizing place field maps over a fixed trajectory rather than by sampling over different trajectories, which is practically much more efficient.

It should be emphasized however, that these and all the subsequent simplifications should not be viewed as limitations of the approach but only as approximations used for simplifying specific computations. The model would also work with more detailed information, e.g., using more precisely estimated spike times or behavioral parameters, physiologically recorded or generated via accurate network models, detailed synaptic transmission mechanisms, etc.

## 3. Overview of the Results

### 3.1. The Learning Region

For a particular set of values (*s, f, N*), a trajectory traversing a place field map $M_{E}$ produces a certain time-dependent coactivity complex T(t). One may inquire whether, and for which ensembles, such a complex acquires the correct topological shape and how long this process may take. As it turns out, the coactivity complexes produced by generic place field maps can assume correct topological shapes, $b_{k}\left(T\right)=b_{k}\left(E\right)$, *k* ≥ 0, in a biologically feasible period—if the spiking parameters fall into a specific domain in the parameter space that we refer to as the *learning region*, L (Figure 1B). It is important to note that although the exact structure of T(t) depends profusely on the details of the map $M_{E}$ (Babichev and Dabaghian, 2018), most large-scale characteristics of T(t), e.g., its Betti numbers, are largely $M_{E}$-independent. This leads to the model's first predictive outcome, namely to the observation that the mean spiking parameters (*s, f, N*) may be used to identify a particular hippocampal “state” with a certain learning capacity,

(1) $$\begin{array}{l} T_{\min}=T_{\min}\left(s,f,N\right). \end{array}$$

The second key observation is that the placement of the learning region in the parameter space matches the biological range of spiking characteristics derived from electrophysiologically recorded data (Dabaghian et al., 2012). *A priori*, this correspondence is not guaranteed: the region L that emerges from the “homological” computations could have appeared anywhere in the parameter space. However, the fact that the “operational” domain of the topological model appears to match the biological domain, suggests that the topological approach captures actual aspects of the neurophysiological computations taking place in the hippocampal network. In particular, it indicates that the physiological neurons can indeed encode a topological map of space in a biologically feasible time. On the other hand, boundedness of L also shows that spatial selectivity of firing does not, by itself, guarantee a reliable mapping of the environment, despite a widespread belief among neuroscientists to the contrary.

Third, the size and the shape of L reflect the scope of the biological variability that the hippocampus can afford in a given environment: the larger the learning region L, the more stable the map (Figure 1B). Indeed, the model implies that the hippocampus can change its operating state inside L without compromising the integrity of the topological map: if one parameter begins to move outside the learning region, then a successful spatial learning can still occur, provided that compensatory changes of other parameters can keep the neuronal ensemble inside L. This observation allows reasoning about the effects of certain diseases [e.g., Alzheimer's Cacucci et al., 2008; Cohen et al., 2013] or environmental toxins [e.g., ethanol Matthews et al., 1996; White and Best, 2000, cannabinoids Robbe and Buzsáki, 2009] that produce more diffuse place fields, lower place cell firing rates, smaller numbers of active cells and thus may disrupt spatial learning by shifting system's parameters beyond the perimeter of the learning region.

Fourth, the structure of the learning region may also vary with the geometry of the environment, the laboriousness of navigation: the greater the task's complexity, the narrower the range that can sustain learning—as suggested by experimental studies (Nithianantharajah and Hannan, 2006; Fenton et al., 2008; Eckert and Abraham, 2010). Thus, despite the topological nature of the information processing, the place cells are not “agnostic” about the scale and the shape of the navigated space. In fact, it can be shown that maps of large spaces can be assembled from the maps of their parts, e.g., if a domain E is split into two subdomains $E_{1}$ and $E_{2}$ that meet but do not overlap, then one can compute the individual learning times $T_{\min}\left(E_{1}\right)$ and $T_{\min}\left(E_{2}\right)$ using only the spikes fired within each subdomain. The sum of these learning times is similar to total time spent by rat in the entire arena, $T_{\min}\left(E\right)\approx T_{\min}\left(E_{1}\right)+T_{\min}\left(E_{2}\right)$, with statistically insignificant differences (Arai et al., 2014). Mathematically, this result may be viewed as an adaptation of the Mayer-Vietoris theorem that states that if a space E is split into pieces $E_{1}$ and $E_{2}$ that overlap over a domain with vanishing homologies, $H_{q}\left(E_{1}\cap E_{2}\right)=0$, then the homologies of the whole space are given by the direct sum of the homologies of the components, $H_{q}\left(E\right)=H_{q}\left(E_{1}\right)⊕H_{q}\left(E_{2}\right)$ (Hatcher, 2002). In case of the coactivity complexes, simulations demonstrate that persistent loops that represent topological obstacles in two complementary domains combine into the set of the persistent loops that represent the whole space, providing a novel perspective on the learning process.

These outcomes of the model correspond well with our subjective learning experiences: the complexity of the task and the size of the navigated environment influence learning time; difficult tasks are accomplished at or just beyond the limits of our capacity; disease or intoxication can reveal limits in our spatial cognition that would normally be compensated for, and so forth.

### 3.2. Coactivity Window

The results discussed above are based on topological analyses of spiking data produced by large populations of coactive place cells; but what defines neuronal coactivity in the first place? At a phenomenological level, an instance of coactivity may be characterized by the length of the period allocated for detecting the spikes fired by two or more cells. Experimental studies suggest that the “physiological” width *w* of the coactivity window ranges between tens to hundreds of milliseconds, with the standard estimate *w* ~ 200 ms (Ang et al., 2005; Huhn et al., 2005; Maurer et al., 2006; Mizuseki et al., 2009). The topological model allows addressing this question theoretically: one can ask, e.g., what range of window sizes *could* allow constructing topological maps and would these values match the biological range of coactivity periods? One can also inquire, given a particular width *w*, whether the dynamics of T(t) depends on a specific arrangement of the coactivity intervals along the time axis and how sensitive the results may be with respect to the windows' variations from one instance of coactivity to another. In biological terms: can the noise and/or variability of coactivity readouts affect the animal's learning capacity?

As it turns out, the answer to the latter two questions is negative: the statistics of place cell coactivity and hence the structure of the coactivity complex do not exhibit strong dependence on either the coactivity windows' random temporal shifts or on the window sizes' “jitter” (both for up to 50% of the mean *w*). On the one hand, this justifies using a single parameter *w* for studying the dependence of the coactivity complex' structure on the window width. On the other hand, it is clear that learning dynamics should depend on the systematic changes of *w*: if the coactivity window is too narrow, then the spike trains produced by the place cells will often “miss” one another, so that the map will either fail or take a long time to emerge. However, if *w* is too wide, then the place cells with disconnected place fields will contribute spurious links that may compromise the map's structure.

**Figure 2:**
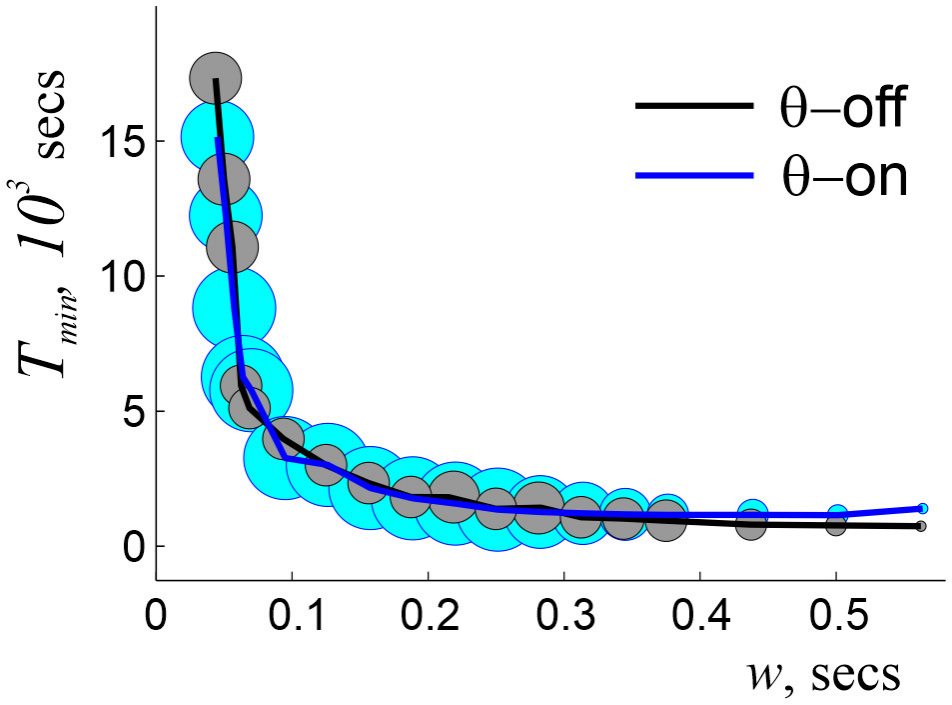
Dependence of learning time on window width, with (blue line) and without (black line) θ-modulation of spiking activity. The radius of the circles indicates the percentage of times when topological learning is successful. In both cases, the coactivity complexes with physically correct topological shapes start to form at about *w*_*o*_ = 0.2 θ-periods, when the learning time is long (hours) and sensitive to the variations of *w*, and fail at *w* ~ 4.5 θ-periods, when learning becomes unreliable. At *w*_*s*_ ~ 1.5 θ-periods the dependence *T*_min_(*w*) plateaus, marking the domain of stable *w*, which continues to *w*_*s*_ ~ 3 θ-periods. As *w* grows further, the success rate diminishes, and for *w*_*s*_ > 5.5 θ-periods topological learning fails. Here *s* = 23 cm, *f* = 28 Hz, and *N* = 350 cells.

Simulations show that indeed, an accurate topological map emerges within a well-defined range of *w*s, *w*_*o*_ ≈ 25 ≤ *w* ≤ *w*_*c*_ ≈ 1, 250 ms, beyond which the maps have vanishing convergence rates (i.e., maps rarely or never produces the correct Betti numbers). In-between, the learning time follows a power law dependence, $T_{\min}\left(w\right)\sim w^{-\alpha }$, with α ≈ 1.2 starting at high values [*T*_min_(*w*_*o*_) ≈ 5 h] that rapidly decrease with growing *w* (Figure 2). The “operational” range of *w*s is even smaller since the biological dependence *T*_min_(*w*) should be not only finite, but also *stable*, i.e., it should not be hypersensitive to variations of *w* or exhibit low convergence rates. In the model, such a range of *w*s lays approximately between 125 and 250 ms (Figure 2), which matches the domain implicated in experimental studies. Thus, the model once again allows deriving the physiologically observed values—in this case the operational widths of the coactivity windows—from purely theoretical considerations.

### 3.3. The Brain Waves

The temporal organization of the spike trains is strongly influenced by the oscillating extracellular electrical fields—the *brain waves*, that control the temporal architecture of the spiking activity and the parcellation of the information flow in the brain (Buzsáki and Draguhn, 2004). In particular, the θ-wave (4–12 Hz) and the γ-waves (40–80 Hz), are known to modulate the place cells' activity at several timescales and affect spatial leaning (Buzsáki, 2002; Hasselmo et al., 2002; Colgin and Moser, 2010). However, it remains unclear at what level, and through what mechanisms, do these waves exert their influence. Most theoretical analyses address the effect of θ- and γ-rhythms on individual cells' spiking (Lisman and Idiart, 1995; Jensen and Lisman, 2000; Hasselmo et al., 2002). In contrast, the topological model allows addressing this question at the ensemble level, by tracing how the θ- and the γ-modulation of spike trains changes the dynamics of the corresponding coactivity complexes, e.g., the speed of their convergence toward correct topological shape, the statistics of topological defects exhibited during this process and so forth. Let us discuss a few examples.

**Figure 3:**
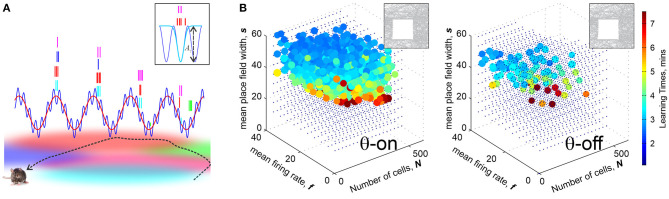
Brain waves enhances learning. **(A)** θ-precession and γ-synchronization modulate place cell spiking activity: Spike times precess with the θ-rhythm (≈8 Hz), schematically shown by the red wave: as the rat traverses a place field, the corresponding place cell discharges at a progressively earlier phase in each new θ-cycle. The preferred θ-phases correspond to γ-cycles (≈60 Hz). The blue wave shows the net θ+γ amplitude. Boxed image: the spikes, shown by tickmarks colored according to the place fields traversed by the animal's trajectory, cluster over the γ-troughs, yielding dynamical cell assemblies. The spike time probabilities are modulated by a Boltzmann factor $e^{-A_{\gamma }\left(t\right)/\tau }$, where *A*_γ_ the amplitude of a trough and τ is an “effective temperature.” **(B)** Learning regions with θ-precession (left) and without it (right). In the latter case, the size and the density of L diminishes, indicating that θ-oscillations enhance place cells' ability of to encode spatial maps, making them more resilient in the face of the spiking rate or population size changes. Computations are made for a 1 × 1 m environment shown in the top left corner.

(i) θ-*phase precession* is a key mechanism by which the θ-wave controls place cell's spiking: as a rat moves through a place field, the corresponding place cell spikes near a certain preferred θ-phase that progressively diminishes for each new θ-cycle (Buzsáki, 2005; Huxter et al., 2008) (Figure 3A). As discussed in Jensen and Lisman (1996) and Skaggs et al. (1996), this phenomenon helps to recapitulate the temporal sequence of the rat's positions in space during each θ-period and it is therefore widely believed to enhance learning (Buzsáki, 2002, 2005).

Simulations show that indeed, θ-precession significantly enlarges the learning region, making otherwise poorly performing ensembles much more capable of learning. Without θ-precession, the learning region L is small and sparse, and vice versa, certain place cell ensembles that in absence of θ lay beyond the learning region, become functional with the addition of θ-precession (Figure 3B). Moreover, θ-precession increases the probability of the correct outcome for ensembles that occasionally fail to form an accurate map, which suggests that θ-precession may not just correlate with, but actually enforce spatial learning (Arai et al., 2014).

In terms of the coactivity complex' structure, θ-enhancement of learning is manifested through shortened durations of the spurious 1*D* cycles, while initially increasing their number. In other words, θ-modulation suppresses spurious defects in the cognitive map at the price of creating more transient errors at the initial stages of the navigation. Curiously, simulations also show that learning times are relatively insensitive to the details of the θ-wave structure: the presence of a spike-modulating θ-rhythm by itself is more important than a specific wave shape (Arai et al., 2014).

As for the interplay with the coactivity parameters, the stabilization of the *T*_min_(*w*) dependence is achieved at approximately the same range of *w*s as without the θ-precession, at *w* ~ 1 − 2θ-cycles (Arai et al., 2014) (Figure 2). Such recurrent matches between the preferred coactivity timescale and the θ-timescale suggest that the interplay between neuronal spiking and the parameters of animal's behavior (e.g., speed) required for optimal processing of topological information may actually define the temporal domain of neuronal synchronization in the rat's hippocampal network. Thus, θ-modulated coactivity complexes provide a self-consistent description of the hippocampal network's function at the θ-timescale, predicting *inter alia* an optimal integration window for reading out the information and the temporal domain of synchronization.

(ii) γ-*modulation of spiking*. As *w* shrinks beyond the range predicted for the independently θ-precessing place cells (*w* < *w*_*o*_), spatial learning fails. Interestingly, this happens precisely at the timescale where complementary mechanisms of spike synchronization, driven by the second key component of the hippocampal brain waves—the γ-oscillations—are taking over (Colgin et al., 2009; Buzsáki and Wang, 2012). This raises question about whether an additional γ-synchronization of spiking could improve the predicted properties of the cognitive map, i.e., produce topologically correct coactivity complexes.

Physiologically, γ-wave represents fast oscillations of the inhibitory post-synaptic potentials. As its amplitude *A*_γ_(*t*) drops at a certain location, the surrounding cells with high membrane potential spike (Lisman, 2005; Jia and Kohn, 2011; Nikoli et al., 2013). As a result, each γ-trough defines the preferred θ-phase of several cells, i.e., marks an ignition of a particular place cell combination, represented by a coactivity simplex. Computationally, coupling spike times with the γ-wave can be achieved by modulating neuronal firing rates with a Boltzmann factor $e^{-A_{\gamma }\left(t\right)/\tau_{i}}$. The parameter τ_*i*_ can be interpreted as an effective “temperature” that controls the temporal spread of spikes around the *i*th γ-trough: for large mean τ = 〈τ_*i*_〉, the spikes are “hot,” i.e., spread diffusely near the γ-troughs and for small τ they “freeze” at them. In particular, the case in which the spike trains are uncorrelated with the γ-troughs corresponds to the limiting case of an “infinitely hot” hippocampus (τ = ∞, e.g., the pure θ-modulated cells discussed above). Meanwhile, the “physiological” effective temperature that describes the characteristic huddling of spikes within a γ-period observed in the experiments (Colgin et al., 2009; Colgin and Moser, 2010) is comparable to the mean γ-amplitude, $\tau \approx \bar{A_{\gamma }}$.

The net effect of the γ-modulation on the coactivity complexes is as follows: as the effective temperature drops and the temporal spread of the spikes near the γ-troughs shrinks, the coactivity complexes produce fewer, faster-contracting spurious loops. In particular, at the “physiological” effective temperatures, γ-synchronized cognitive map can robustly capture the topology of the environment by integrating place cell coactivity at the γ-timescale, i.e., yield *finite* learning times at *w* < *w*_*o*_s, which provides a direct demonstration of the importance of the γ-synchronization at the systemic level.

This result may shed light on the well-known correlation between successful learning and retrieval with the increase of the γ-amplitude in raised attention states (Moretti et al., 2009; Vugt et al., 2010; Lundqvist et al., 2011; Trimper et al., 2014). In particular, it helps understanding why suppression of the γ-waves induced, e.g., by psychoactive drugs (Whittington et al., 2000a,b), such as cocaine (Dilgen et al., 2013; McCracken and Grace, 2013), or arising due to neurodegeneration or aging (Vreugdenhil and Toescu, 2005; Lu et al., 2011), usually correlates with learning impairments—according to the model, all these phenomena suppress map formation—or retrieval—at the γ timescale. On the constructive side, the model suggests a new characteristics of the γ-synchronized spiking activity—the effective γ-temperature of spiking—that may be studied empirically and explained via neuronal mechanisms.

### 3.4. Ramifications of Coactivity Complexes

The predictions derived from the constructions discussed above are not universal. For example, a direct application of the model to the case of the bats navigating 3*D* caves (Ulanovsky and Moss, 2007; Yartsev and Ulanovsky, 2013) often produces dysfunctional coactivity complexes, with hundreds of persistent spurious loops—even for the experimentally observed parameters of spiking activity (Hoffman et al., 2016). On the one hand, this failure can be explained by the relatively high speeds of the bat's movements (over 2 m/s), which allows producing spurious coactivities between place cells with non-overlapping place fields (Hoffman et al., 2016). On the other hand, it also suggests that the very idea that place cells operate by responding to certain spatial domains (currently dominating in the field) may be only a simplified interpretation of their spiking mechanism, suitable for low speeds and basic environments. The model points out that deriving topological maps from such “passive responses” may, at higher speeds, generate mismatches between the spatial pattern of the prearranged place fields and the temporal pattern of the corresponding place cells' coactivities. In other words, the model suggests that the raw pool of place cell spiking data requires *editing*—a surprising conclusion because it appeals to reasoning beyond the model's original setup. In effect, it suggests that the hippocampal network should be *wired* to highlight some place cell coactivities and suppress others, even though no explicit references to the networks' structure were made in the original Alexandrov-Čech construction.

**Figure 4:**
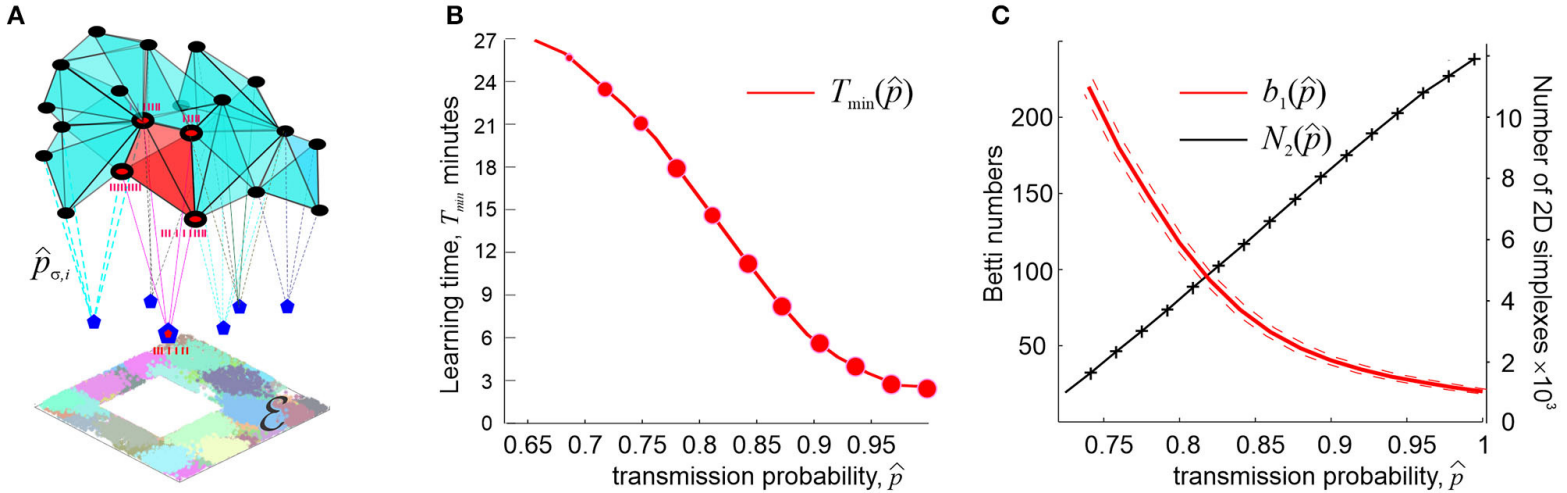
Synaptic efficacies and cell assembly complexes. **(A)** Cell assemblies are functionally interconnected network of place cells (black dots) are modeled as cliques of the coactivity graph G. Spikes from *k*th pair of coactive place cells in an assembly σ are transmitted to a readout neuron (blue pentagons) with probability *p*_σ, *k*_ < 1 (an ignited cell assembly is shown in red). The net structure of the cell assembly network is represented by the corresponding cell assembly complex $T_{CA}$, which captures the topology of underlying environment E. **(B)** The number of coactivity links shrinks with the diminishing spike transmission probability at a power rate (black line), whereas the number of spurious topological loops in $T_{CA}$ proliferates exponentially. **(C)** As synapses weaken, the learning time *T*_min_ grows at a power rate. The size of the data points represents the percentage of the outcomes with the correct Betti numbers [$b_{0,1}\left(T_{CA}\right)=b_{0,1}\left(E\right)=1$]. Computations are performed using an ensemble of *N* = 400 neurons with a mean firing rate of *f* = 28 Hz and mean place field size 30 cm.

Curiously, this line of arguments addresses to a well-known neurophysiological phenomenon, namely the fact that place cells tend to form operative units known as *cell assemblies*—functionally interconnected groups of neurons that drive their respective “readout” neurons in the downstream networks (Harris et al., 2003; Harris, 2005; Jackson and Redish, 2007; O'Neill et al., 2008; Buzsaki, 2010). The spiking response of the latter actualizes connectivity relationships between the regions encoded by the individual place cells: if a specific instance of place cell coactivity does not elicit a response of a readout neuron, then the corresponding connectivity information does not contribute to the hippocampal map (Buzsaki, 2010; Babichev et al., 2016b). A cell assembly network of a specific architecture can thus control processing of the information supplied by the place cell spiking activity and the overall connectivity structure of the cognitive maps (Figure 4A).

#### 3.4.1. Clique Coactivity Complexes

A simple model a place cell assembly network can be built by constructing a coactivity graph G, whose vertexes *v*_*i*_ correspond to place cells *c*_*i*_ and the links, ς_*i*_0_*i*_1__ = [*v*_*i*_0__, *v*_*i*_1__] represent the connections (functional or physiological) between pairs of coactive cells (Burgess and O'Keefe, 1996; Muller et al., 1996). The place cell assemblies then correspond to fully interconnected subgraphs of G, i.e., to its maximal cliques ς = [*c*_*i*_0__, *c*_*i*_1__, …, *c*_*i*_*n*__]. As a combinatorial objects, cliques are identical to the simplexes span by the same sets of vertexes; hence the collection of G-cliques produces a complex (Jonsson, 2008) that may serve as a schematic representation of either the cell assembly network or the cognitive map encoded by it (Babichev et al., 2016a).

Simulations show that such complexes, denoted below as $T_{\varsigma }$, are structurally very similar to the original coactivity complexes derived from the higher-order place cell coactivities, which we will denote as $T_{\sigma }$. However functionally, $T_{\varsigma }$s often perform much better than $T_{\sigma }$s, e.g., they exhibit a much smaller number of shorter-living spurious loops, more robust learning times, etc. (Babichev et al., 2016a; Basso et al., 2016; Hoffman et al., 2016). The explanation for this effect is simple: the lowest order, *pairwise* place cell coactivities are captured easier and more reliably than the higher-order coactivity events (Katz et al., 2007; Brette, 2012). An additional advantage is offered by a structural flexibility of the clique coactivity complexes, since it is possible to assemble its individual cliques $\varsigma \in T_{\varsigma }$ by *accumulating* low order coactivities over time, rather than by *detecting* higher-order coactivity events. For example, in order to identify a third-order coactivity clique, ς = [*c*_*i*_0__, *c*_*i*_1__, *c*_*i*_2__], one can first detect the coactive pair [*c*_*i*_0__, *c*_*i*_1__], then [*c*_*i*_1__, *c*_*i*_2__] and then [*c*_*i*_0__, *c*_*i*_2__], over an extended integration window ϖ, whereas in order to produce a coactivity simplex σ = [*c*_*i*_0__, *c*_*i*_1__, *c*_*i*_2__], all three cells must become active within a single coactivity window *w*.

From the physiological perspective, the clique construction can be used to model a wide scope of physiological phenomena, e.g., for testing whether the readout neurons may operate as “coincidence detectors” that respond to nearly simultaneous activity of the pre-synaptic cells [for short integration windows ϖ ~ *w* Katz et al., 2007; Brette, 2012] or as “integrators” of the spiking inputs [for ϖ ≫ *w* König et al., 1996; Magee, 2000; London and Häusser, 2005; Spruston, 2008; Ratté et al., 2015], along with the intermediate and/or mixed cases. The original approach based on the Alexandrov-Čech's construction corroborates with the first scenario: indeed, the nerve complex N is derived from the spatial overlaps between the regions, which mark the domains of nearly simultaneous place cell coactivity. The architecture of the clique coactivity complex suggests an alternative approach that significantly broadens the models' capacity to represent synaptic computations.

Simulations show that, in fact, the connections within most cliques of G activate nearly simultaneously, i.e., most simplexes of $T_{\sigma }$ are also present in $T_{\varsigma }$. Nevertheless, there exists a small population of cliques that are never observed as simultaneous coactivity events and require assembling over extended periods (Hoffman et al., 2016). As a result, clique coactivity complexes $T_{\varsigma }$ are typically larger and produce much fewer spurious topological loops that rapidly disappear with learning. In particular, such complexes produce correct topological maps of 3*D* spaces for the experimentally observed parameters of the spiking activity (Hoffman et al., 2016), suggesting that the readout neurons in bats' (para)hippocampal areas should function as integrators of synaptic inputs (with estimated spike integration period of about 4 min), rather than detectors of place cells' coactivity—a prediction that may potentially be verified experimentally.

Another curious difference between the rats' and the bats' cognitive map construction mechanism is that less than 4% of the bat's place cells exhibit significant θ-modulated firing (Yartsev and Ulanovsky, 2013), which implies that θ-precession in these animals may not play the same role as in rats. Indeed, simulating bat's movements with and without θ-precession reveals that in the θ-off case, the ensembles of place cells acquire correct maps faster than in the θ-on cases, producing fewer topological loops both in the simplicial and in the clique coactivity complexes (Hoffman et al., 2016). To explain these results, one can consider the effect of θ-precession from two perspectives: on the one hand, it synchronizes place cells and hence increases their coactivity rate, which may help learning (Buzsáki, 2002; Harris et al., 2002; Lee et al., 2004; Geisler et al., 2010; Jezek et al., 2011). On the other hand, it can be viewed as a constraint that reduces the probability of the cells' coactivity and hence decimates the pool of coactivity events (Skaggs et al., 1996). In relatively slow moving rats, when the coactivity events are reliably captured, the first effect dominates, contributing a steady influx of grouped spikes to downstream neurons. In rapidly moving bats however, when the network struggles to capture the coactivities, the constraint imposed by phase precession acts more as an impediment and slows down spatial learning process.

### 3.5. Cell Assembly Complex

Question arises, whether the coactivity complexes may be implemented, in some capacity, in physiological networks or vice versa, whether it is possible to construct complexes that capture the organization of the cell assembly networks. Simulations show that the original set of coactive place cell combinations is very large: the numbers of *d*-dimensional simplexes in $T_{\varsigma }$, *N*_*d*_, scale proportionally to the binomial coefficients $C_{N}^{d+1}$. More specifically, it can be shown that the ratios $\eta_{d}=N_{d}/C_{N}^{d+1}$ depend primarily on the mean place field sizes and the firing rates and not on the number of cells within the ensemble, *N* (Arai et al., 2014). In contrast, the number of cells that may potentially serve as readout neurons is similar to the number of place cells *N*, which implies that only a small fraction of coactive place cell groups can form assemblies (Shepherd, 2004; Buzsaki, 2010). This raises the question: is it possible to identify a sufficiently small but functionally complete set of place cell combinations—putative cell assemblies—using simple selection rules?

In model's terms, the task of identifying a subpopulation of coactive place cell combinations corresponds to selecting a “cell assembly subcomplex” $T_{CA}$ of $T_{\varsigma }$, according to some biologically motivated criteria. First, the total number of the maximal simplexes in $T_{CA}$ should be comparable to the number of its vertexes (i.e., of active cells), $N_{\max}\left(T_{CA}\right)\approx N(T_{CA}$), but the latter should not differ significantly from the original number of place cells, $N\left(T_{CA}\right)\approx N\left(T_{\varsigma }\right)$. Second, only a few cell assemblies (selected cliques) should be active at a given location, to avoid redundancy of the place cell code. Conversely, the periods during which all place cell assemblies are inactive should be short, so that the rat's movements should not go unnoticed by the hippocampal network. Third, the larger is the number of cells shared by consecutively igniting cell assemblies (i.e., by the adjacent simplexes in a simplicial path), the more contiguous is the representation of the rat's moves. Hence the contiguity between the simplexes in $T_{CA}$ should not decrease compared to $T_{\varsigma }$. Lastly, $T_{CA}$ should correctly capture the topological shape of the environment (Babichev et al., 2016a).

As it turns out, it is possible to carry out the required construction by selecting the most prominent combinations of coactive place cells—the ones that appear most frequently. This selection principle is motivated by the Hebbian “fire together wire together” neuronal plasticity mechanisms: frequently appearing combinations have a higher chance of being wired into the network (Neves et al., 2008). Specifically, one can construct the desired clique complexes by identifying the connections the coactivity graphs G(ξ) that activate at a rate exceeding a certain threshold ξ. Alternatively, one can select, for every cell *c*_*i*_, its *n*_0_ neighbor-cells that are most frequently coactive with *c*_*i*_, which yields another family of coactivity graphs, $G\left(n_{0}\right)$. Computations show that the first family, G(ξ), exhibits certain random graph properties while the second family, $G\left(n_{0}\right)$, demonstrates scale-free properties (Barabási and Albert, 1999; Albert and Barabási, 2002), characteristic of the hippocampal network (Bonifazi et al., 2009; Li et al., 2010). However, both families of “restricted” coactivity graphs allow constructing operational cognitive map models, for a viable set of ξs and *n*_0_s.

As expected, the size and the dimensionality of the corresponding clique complexes, $T_{\varsigma }\left(\xi \right)$ and $T_{\varsigma }\left(n_{0}\right)$, decrease with the growing threshold ξ or diminishing *n*_0_. In addition, their maximal simplexes become more contiguous and their number, *N*_max_, remains close to the number of cells. Lastly, the topological behavior of both $T_{\varsigma }\left(\xi \right)$ and $T_{\varsigma }\left(n_{0}\right)$ is also regular: with minor rectification algorithms that do not change significantly the complex's structure or alter the appearance rate of simplexes, correct topological shapes can be attained as fast and as reliably as with the entire set of the place cell coactivities, without compromising the place cell coverage of the environment or fragmenting the map (Babichev et al., 2016a). Thus, the generic biological requirements listed above are met and we may conclude that the selected “critical mass” of coactive place cell combinations can produce viable cell assembly complexes $T_{CA}\left(\xi \right)$ and $T_{CA}\left(n_{0}\right)$ (Babichev et al., 2016a).

### 3.6. Synaptic Parameters

The physiologically implementable cell assembly complexes $T_{CA}$ set the stage for further developments of the topological model. For example, the simplexes of $T_{CA}$ can be rigged with parameters describing transferring, detecting and interpreting neuronal (co)activity in the corresponding cell assemblies, allowing us to account for the effects of the hippocampal network's synaptic architecture and providing a basic description of the synaptic computations in the cell assemblies.

In a phenomenological approach, synaptic connections can be characterized simply by the probabilities of transmitting spikes from a place cell to a readout neurons' membranes and by the probabilities that the latter will spike upon collecting their inputs. If the cell assemblies are modeled as cliques of the coactivity graph, then the key role is played by the probability of transmitting the coactivity from the pairs of coactive place cells to the corresponding readout neurons' and response probabilities. In principle, these probabilities can be evaluated using detailed neuronal and synaptic models; however, in a simpler phenomenological approach, they may be regarded as random variables drawn from stationary, unimodal distributions with the modes *p*_∗_ (transmission) and *q*_∗_ (response) and the variances Δ_*p*_ and Δ_*q*_. The stationarity here implies that we disregard synaptic plasticity processes (Brunel et al., 2004; Barbour et al., 2007; Buzsáki and Mizuseki, 2014).

Under such assumptions, it is possible to study how the large scale, systemic characteristics of the spatial memory map depend on the synaptic strengths, at what point spatial learning may fail, and so forth. It can be shown, e.g., that if the characteristic coactivity transmission probability is high (0.9 ≤ *p*_∗_ ≤ 1) then its small variations do not produce strong effects on the spatial map. On the other hand, as *p*_∗_ decreases further, the changes accumulate and, as *p*_∗_ approaches a certain critical value *p*_*crit*_, learning times diverge at a power rate,

$$\begin{array}{l} T_{\min}\propto {\left(p_{*}-p_{crit}\right)}^{-\kappa }, \end{array}$$

with κ ranging typically between 0.1 and 0.5 (Figure 4B). The effects produced by the diminishing probability of the post-synaptic neurons' responses, *q*_∗_, are qualitatively similar but weaker than the effects of lowering the spike transmission probability *p*_∗_ (Dabaghian, 2019).

These results suggest explanations for numerous observations of correlative links between weakening memory capacity and deterioration of synapses, broadly discussed in neuroscience literature (Shapiro, 2001; Selkoe, 2002; Toth et al., 2012). According to model, weakening synapses reduce the size the coactivity complex and degrade its topological structure. For example, simulations demonstrate the number of connections in the coactivity graph G near *p*_*crit*_ drops as $N_{2}\propto {\left(p_{*}-p_{crit}\right)}^{\delta }$, δ ~ 1, whereas the number of longer-lasting 1*D* spurious loops in the corresponding coactivity complex grows exponentially, log(*b*_1_) ∝ (*p*_*crit*_ − *p*_∗_) (Figure 4C), suggesting a phase transition from a regular to an irregular state (Donato et al., 2016). In physiological terms, this implies that synaptic depletion reduces the number of detectable coactivities while generating defects in the cognitive map, which results in a rapid increase of the learning time.

Moreover, weakening synapses reduce the learning region down to its compete disappearance at *p*_∗_ = *p*_*crit*_, which suggests that spatial learning may fail not only because the parameters of neuronal firing are pushed beyond a certain fixed “working range,” but also because that range itself may shrink or cease to exist. In particular, the fact that the learning region disappears if the transmission probability drops below the critical value implies that deterioration of memory capacity produced by the synaptic failure cannot be compensated by increasing the place field's firing rates or by recruiting a larger population of active neurons—for more details see Dabaghian (2019).

### 3.7. Dynamical Cell Assemblies

**Figure 5:**
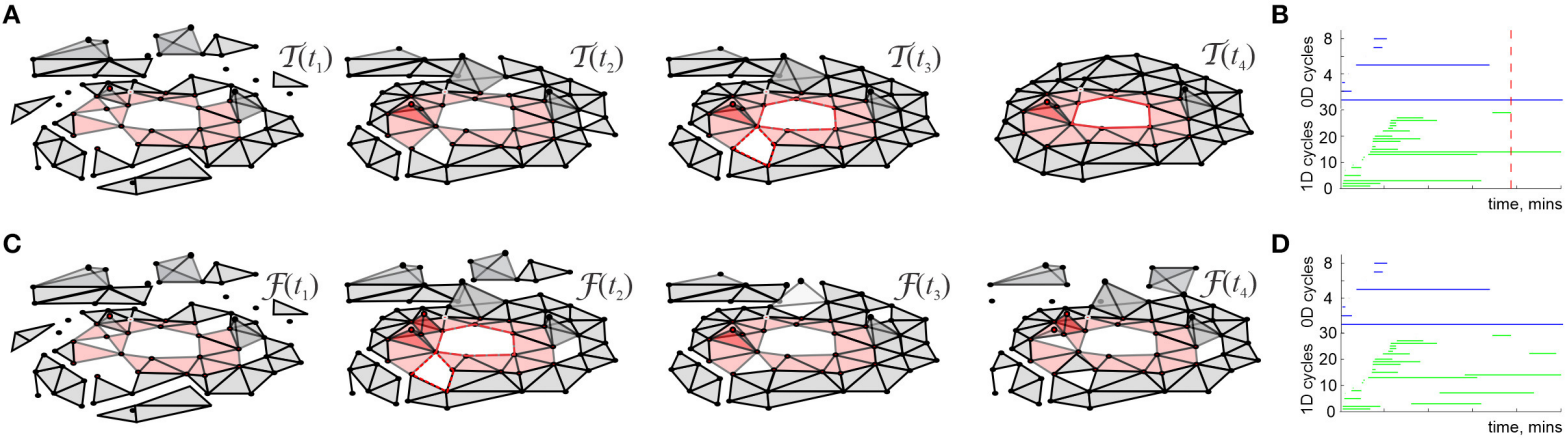
Perennial and flickering coactivity complexes. **(A)** In absence of decay, the coactivity complex T steadily grows. At first, it contains many pieces riddled transient holes, but then, as the place cells' spiking information accumulates, there emerges just one stable piece and only one hole survives (shown by red chain of simplexes)—the one that corresponds to non-contractible simplicial path encircling a hole in the environment E (Figures 3B, 4A). **(B)** The timelines of 0*D* (top) and 1*D* (bottom) topological loops in T, computed using Persistent Homology theory methods, show that the topological shape of the coactivity complex stabilizes. One persistent loop in each dimension remaining after the minimal learning time *T*_*min*_ (vertical dashed line) indicate the stable topological barcode of T. **(C)** If decay is allowed, then the coactivity simplexes may not only appear but also disappear, yielding a “flickering” coactivity complex F. Unlike the perennial complexes T, flickering complexes F may never stabilize, i.e., transient topological defects, described by Zigzag Persistent Homology theory **(D)** may persist indefinitely.

Physiologically, cell assemblies are *dynamic* structures: they may form among the cells that demonstrate repeated coactivity and disband as a result of deterioration of synaptic connections, caused by reduction or cessation of spiking, then reappear during a subsequent surge of coactivity, disband again and so forth (Harris et al., 2003; Buzsaki, 2010). In the model, the formation and disbanding of the cell assemblies is represented by the appearances and disappearances of the corresponding simplexes, so that the net dynamics of the cell assembly network and the evolution of the resulting cognitive map is represented by a “flickering” cell assembly complex, denoted as F(t). Unlike its “perennial” counterpart T(t), which can only grow and stabilize with time (Figures 5A,B), the flickering complex F(t) may inflate, shrink, fragment into pieces that may fuse back together, produce transient holes, fractures, gaps, and other dynamic “topological defects” (Figures 5C,D).

One of the key questions that can be addressed by the model is the following: experimentally, cell assemblies' lifetimes range between minutes (Goldman-Rakic, 1995; Billeh et al., 2014; Hiratani and Fukai, 2014) and hundreds of milliseconds (Whittington et al., 2000a,b; Bi and Poo, 2001; Bennett et al., 2018), whereas cognitive representations of environments can last for days and months (Clayton et al., 2003; Brown et al., 2007; Meck et al., 2013). How can a rapidly rewiring network sustain stable representations of the world? In model's terms, can the large-scale topological properties of F(t) be stable, despite rapid recycling of its simplexes? Computationally, this question can be addressed using Zigzag Persistent Homology theory (Edelsbrunner et al., 2002; Carlsson et al., 2009; Carlsson and Silva, 2010).

*(i) Decaying flickering coactivity complexes*. Flickering of the coactivity complexes and their topological dynamics can be simulated in many ways (see Battiston et al., 2020 for a broad review). A simple model can be based on the dynamics of links of the coactivity graph G as follows.

- Vertexes ς_*i*_ of G appear at the moment of the corresponding place cells' first activation and thereupon remain stable, as place cells do in learned environments (Thompson and Best, 1990).
- A link ς_*ij*_ between vertexes ς_*i*_ and ς_*j*_ appears with probability $p_{ij}^{+}=1$ at the moment when cells *c*_*i*_ and *c*_*j*_ become coactive and disappears with probability $p_{ij}^{-}\left(t\right)=\sim e^{-t/\tau_{ij}}$, where time *t* is counted from the moment of ς_*ij*_'s last activation and τ_*ij*_ defines its *proper* decay time. Below we consider a simple case in which all connections decay at the same rate, τ_*ij*_ = τ,
  (2) $$\begin{array}{l} p_{ij}^{-}\left(t\right)=\sim e^{-t/\tau }, \end{array}$$
  so that the decay dynamics of the flickering coactivity graph depends on a single parameter τ.
- The behavior of the higher-order cliques and hence of the flickering complex $F_{\tau }$ are also defined by the link decay period τ. Note that pairs of place cells may coactivate before decaying, i.e., links in $G_{\tau }$ can rejuvenate; hence cliques of orders *m* ≥ 1 may acquire *effective lifetimes* $\tau_{e}^{\left(m\right)}>\tau$.

As mentioned in §1 of this section, details of the coactivity complex' dynamics depend on the sequence in which the rat traverses place fields in a map $M_{E}$. For a given map $M_{E}$, a trajectory γ(*t*) and fixed physiological parameters (firing rates, place field sizes, etc.), the Betti numbers $b_{k}\left(F_{\tau }\left(t\right)\right)$ depend primarily on the links' decay time τ (Babichev and Dabaghian, 2017a,b; Babichev et al., 2018, 2019). One would expect that if τ is too small (e.g., if the coactivity simplexes tend to disappear between two consecutive co-activations of the corresponding cells), then the flickering complex should rapidly deteriorate without attaining an adequate topological shape. If τ is too large, then the effect of the decaying connections should be insignificant, i.e., the flickering complex $F_{\tau }\left(t\right)$ should follow the dynamics of its “perennial” counterpart $T\left(t\right)\equiv F_{\infty }\left(t\right)$, constructed for the same spiking parameters. In particular, if the place cells' coactivity complex T(t) assumes the correct topological shape in a biologically viable time $T_{\min}\left(T\right)$, then a similar behavior should be expected from its slowly decomposing counterpart $F_{\tau }\left(t\right)$. For intermediate values of τ, the topological dynamics of $F_{\tau }\left(t\right)$ may exhibit a rich variety of behaviors.

**Figure 6:**
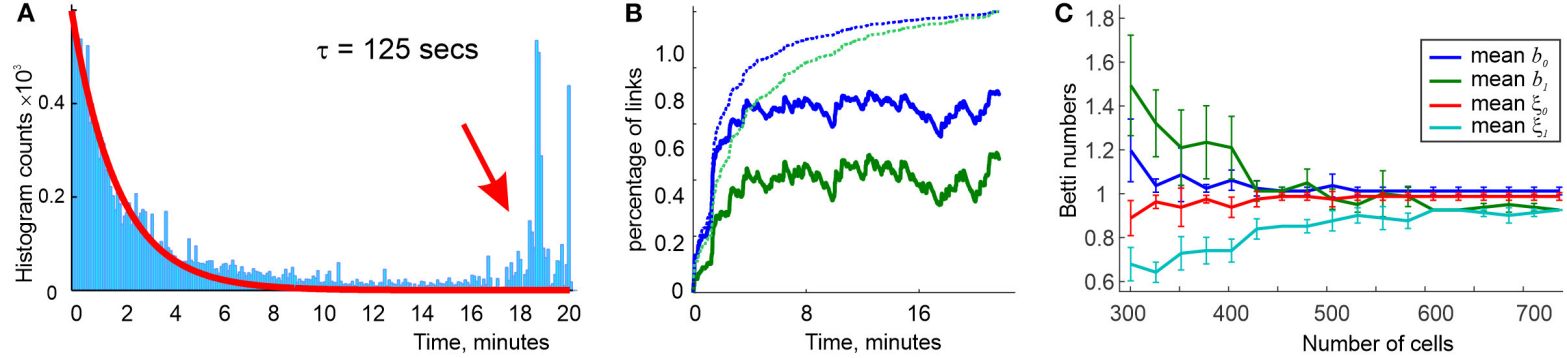
Topological dynamics of the decaying coactivity complex. **(A)** The histogram of the time intervals between the connections' consecutive appearances and disappearances: the lifetimes the rapidly “fluttering” simplexes are distributed exponentially (red line fit). The population of the “survivor” links produces a bulging tail of the distribution (red arrow). **(B)** The population of 1*D* (blue trace) and 2*D* (green trace) simplexes in the decaying “flickering” complex $F_{\tau }\left(t\right)$, compared to the population of 1*D* and 2*D* simplexes in the perennial complex T(t) (dashed lines). The size of $F_{\tau }\left(t\right)$ remains dynamic, whereas T(t) saturates in about 10 min. **(C)** At τ = 125 s decay period, the mean Betti numbers $b_{0}\left(F_{\tau }\right)$ (blue) and $b_{1}\left(F_{\tau }\right)$ (green) converge to their “physical” values *b*_0, 1_ = 1 as the active place cell population increases from *N* = 300 to *N* = 750 units; the *b*_0, 1_-fluctuations decrease (shrinking error bars) and the rates ξ_0, 1_ of producing the correct Betti values grow to nearly 100%. Statistics evaluated over a *T* = 25 min navigation period.

Simulations show that a characteristic interval between successive activations of links in the environment shown on Figures 5A,C is about Δ*t* ≈ 30 s. If the proper decay times are not too large (2.5Δ*t* ≲ τ ≲ 4.5Δ*t*), then the time intervals between consecutive births and deaths of a link ς distribute bimodally: the relatively short lifetimes distribute exponentially, with about twice longer *effective* lifetimes $\tau_{e}^{\left(2\right)}\approx 2\tau$ (higher-order simplexes decay more rapidly, e.g., $\tau_{e}^{\left(3\right)}\approx \tau$, etc.). In addition, there appears a pool of long-living connections that persist throughout the entire navigation period (Figure 6A). In other words, the flickering coactivity complex $F_{\tau }\left(t\right)$ acquires a stable “core” formed by a population of “surviving simplexes,” enveloped by a population of “rapidly fluttering” simplexes.

The resulting mix of skeletal (stable) and fluttering simplexes rapidly grows at the onset of the navigation and begins to saturate by the time a typical link makes an appearance, which, incidentally, is comparable to the “perennial” learning time $T_{\min}\left(T\right)$ (a few minutes). The characteristic size of $F_{\tau }\left(t\right)$ grows to about a half of the size of $F_{\infty }\left(t\right)$, with about 15% fluctuations (Figure 6B). Thus, the population of simplexes in $F_{\tau }\left(t\right)$ is indeed transient: although the size of $F_{\tau }\left(t\right)$ fluctuates slowly from one moment of time to the next, the set of simplexes that are present at a given moment of time *t* but missing at a later moment *t*′, grows as a function of temporal separation |*t* − *t*′|, becoming close to the sizes of either $F_{\tau }\left(t\right)$ or $F_{\tau }\left(t^{\prime }\right)$ in approximately one learning period $T_{\min}\left(T\right)$ (Babichev et al., 2018, 2019).

The topological shape of $F_{\tau }\left(t\right)$ changes much slower: after a brief initial stabilization period, the topological barcode $𝔟\left(F_{\tau }\right)$ remains similar to the barcode of the navigated environment E, exhibiting occasional topological fluctuations at the *T*_min_-timescale (Figure 6C). Thus, the coactivity complex $F_{\tau }$ can preserve not only its approximate size but also its topological structure, despite the ongoing recycling of its simplexes.

**Figure 7:**
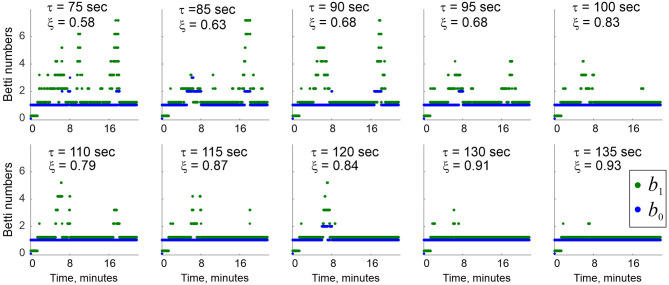
Topological stabilization. As the decay constant τ grows from ~75 to ~135 s, the topological shape of $F_{\tau }\left(t\right)$ stabilizes. A complete suppression of topological fluctuations is achieved for τ ≈ 2−2.5 min, with the other system parameters (rat's speed, place cell firing rates, place field sizes, etc.) within a physiological range. Blue and green dots show Betti numbers *b*_0_ and *b*_1_ at select moments of time. The ξ values show the percentage of times when physically correct topological signature was captured.

As τ grows, the effective lifetimes $\tau_{e}^{\left(2\right)}$ and $\tau_{e}^{\left(3\right)}$, as well as the number of simplexes actualized at a given moment increase approximately linearly, yielding a growing “stable core” (Figure 6). As a result, a *complete suppression* of topological fluctuations in the coactivity complex is achieved at a finite values of τ = τ_∗_ (Figure 7), which gives a theoretical estimate for the rate of physiological transience that permits stable representations of the environment E (Babichev et al., 2018). This observation illustrates the phenomenon of *emergent topological stability* in flickering complexes, which may provide insight into how transient networks sustain lasting representations of stable physical reality.

*(ii) Finite latency flickering coactivity complexes*. An alternative model of flickering clique complexes can be built by restricting the period over which the coactivity graph is formed to a shorter “spike integration” time window ϖ (Theunissen and Miller, 1995; Hoffman et al., 2016; Perea, 2019). In such approach, the coactivity simplexes that emerge within the starting ϖ-period, ϖ_1_, will constitute a coactivity complex $F\left(\varpi_{1}\right)$; the simplexes appearing within the next window, ϖ_2_ will form the complex $F\left(\varpi_{2}\right)$ and so forth. A given clique-simplex ς (as defined by the set of its vertexes) may therefore appear through a chain of consecutive windows, ϖ_1_, ϖ_2_, …, ϖ_*k*−1_, then disappear at the *k*^th^ step ϖ_*k*_ (i.e., $\varsigma \in F(\varpi_{k-1}$), but $\varsigma \notin F\left(\varpi_{k}\right)$), then reappear in a later window ϖ_*l*≥*k*_, then disappear again, and so forth. The duration of ς's existence between its *k*-th consecutive appearance and disappearance, δ*t*_ς, *k*_, can then be as short as the shift between the consecutive windows Δϖ or as long as the animal's navigation session.

**Figure 8:**
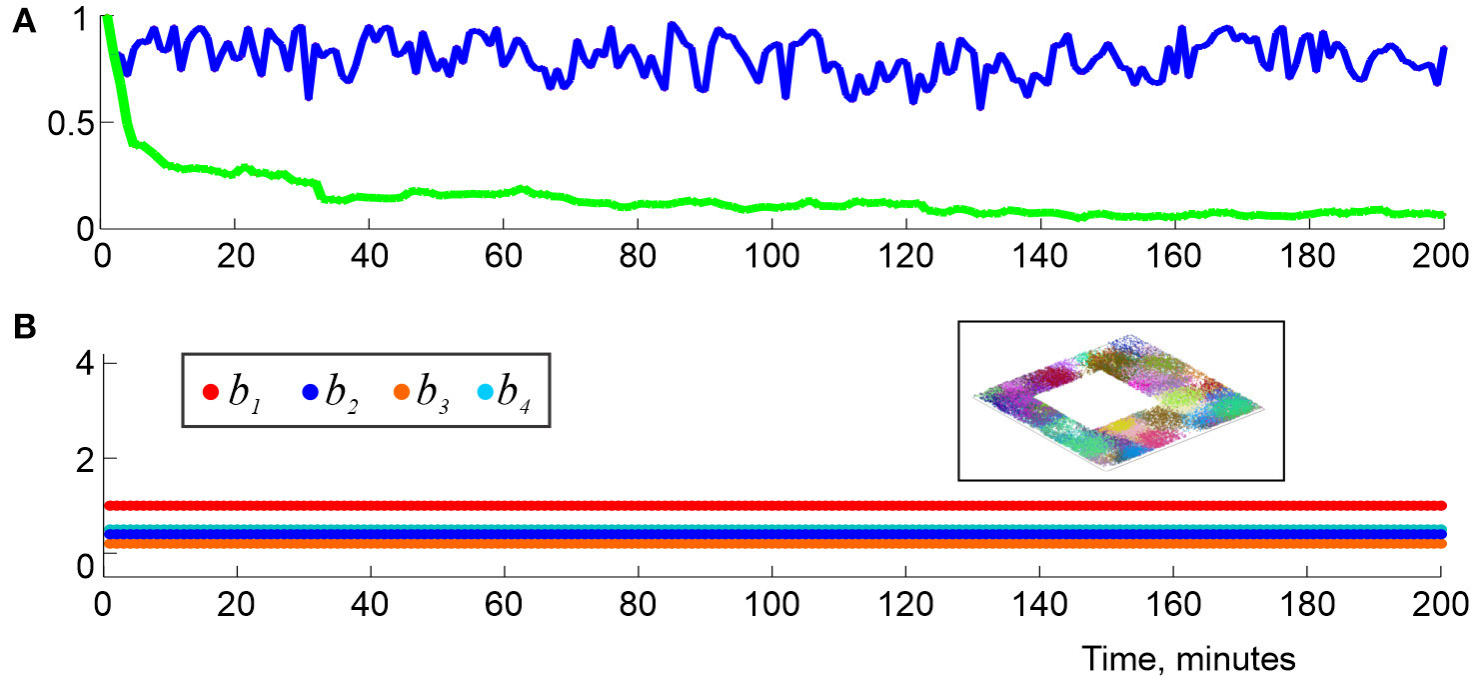
Topological dynamics in the finite latency flickering complexes. **(A)** At each moment, *t*_*n*_, the blue line shows the proportion of maximal simplexes of $F_{\varpi }\left(t_{n}\right)$ that were present at the previous time *t*_*n*−1_, i.e., in $F_{\varpi }\left(t_{n-1}\right)$. The green line shows the proportion of maximal simplexes of $F_{\varpi }\left(t_{n}\right)$ that were present at the onset of the navigation, i.e., in $F_{\varpi }\left(t_{1}\right)$. The latter population changes by about 95% in about 2 min. **(B)** The low-dimensional Betti numbers, *b*_1_, *b*_2_, *b*_3_, and *b*_4_ (colors shown in the left box) as a function of time, computed using ϖ = 1.5*T*_min_ demonstrate full topological stabilization of $F_{\varpi }\left(t\right)$, whose shape fully matches the topological shape of the underlying environment (right box) at all times. Here (*s, f, N*) is (23, 28, 350).

It is natural to view the individual, “instantaneous” complexes $F\left(\varpi_{i}\right)$ as instantiations of a single “finite latency” flickering coactivity complex, $F\left(\varpi_{i}\right)=F_{\varpi }\left(t_{i}\right)$. As it turns out, such complexes exhibit a number similarities with the decaying complexes $F_{\tau }\left(t\right)$, e.g., for $\varpi \geq T_{\min}\left(T\right)$ the pool of maximal simplexes is renewed at about ϖ timescale, but the net number of simplexes contained in $F_{\varpi }\left(t\right)$ changes within about 5−10% of its mean value (Figure 8A). Biologically, this implies that a cell assembly network that described by $F_{\varpi }\left(t\right)$ fully rewires in about a ϖ period, without changing its overall size. Specifically, for ϖ exceeding the perennial learning time $T_{min}\left(T\right)$ and small time steps Δϖ ≳ 0.01ϖ, the intervals δ*t*_ς, *k*_, as well as their means, *t*_ς_ = 〈δ_*t*_ς, *k*_〉*k*_, are exponentially distributed, which allows characterizing the simulated cell assemblies by a half-life, τ_ϖ_ that typically varies within τ_ς_ ≈ 3−20 s. As ϖ widens, the mean lifetimes *t*_ς_ of the maximal simplexes grow, and vice versa, as the memory window shrinks, simplex-flickerings intensify.

On the other hand, the large-scale shape of $F_{\varpi }\left(t\right)$ is much more stable than its individual simplexes, as in the case of the “decay model” (2). The topological fluctuation reduce with growing ϖ, and, for sufficiently long latency periods ϖ ≥ ϖ_∗_ ≈ 1.5 *T*_min_ they tend to *disappear completely* (Figure 8B)—even though the simplexes' lifetimes remain short ($\tau_{\varpi }^{*}\approx 15$ s for the environment illustrated on Figure 5A). For sufficiently long latencies, $\varpi ≳1.2T_{\min}\left(T\right)$, the time required to produce physical barcode $𝔟\left(F_{\varpi }\right)=𝔟\left(E\right)$ within typical window ϖ_*k*_ is similar to the perennial learning time, ${\bar{T}}_{min}={\langle T_{min}^{\left(k\right)}\rangle }_{k}\approx T_{\min}\left(s,f,N\right)$, with a variance of about 20−40% of the mean, which shows that topological dynamics of the simulated cognitive maps is largely time-invariant. In plain words, this result shows that accumulation of the topological information can start at any point (e.g., at the onset of the navigation or after an exploratory delay) and produce the desired stable map after about the same period of learning. In effect, this observation justifies using perennial coactivity complexes for estimating *T*_*min*_ in Dabaghian et al. (2012), Arai et al. (2014), Basso et al. (2016), Hoffman et al. (2016), and Dabaghian (2019).

Moreover, for these latencies [$\varpi ≳\varpi_{crit}\approx 1.4T_{\min}\left(T\right)$], the instantaneous learning times $T_{min}^{\left(k\right)}$ become ϖ-independent, i.e., the finite latency model can provide a *parameter-free* characterization of the time required by a network of place cell assemblies to represent the topology of the environment and establishes the timescale for the topological fluctuations in the simulated cognitive map.

Note that finite latency model (*ii*) cannot be naívely reduced to the decay model (*i*) by fixing the links' lifetimes, i.e., by using the decay probability

(3) $$\begin{array}{l} p_{-}\left(t\right)=\{\begin{array}{cc} 1 & \text{if }t=\tau \\ 0 & \text{if }t\neq \tau . \end{array} \end{array}$$

The topological structure of the “quenched-decay” coactivity complex $F_{\tau }^{*}\left(t\right)$ controlled by the distribution (3) exhibits more unstable dynamics than either $F_{\varpi }\left(t\right)$ or $F_{\tau }\left(t\right)$, even for the τ-values that reliably produce physical Betti numbers for the exponentially distributed lifetimes. As decay slows down (i.e., as τ grows), the population of survivor links produced by (3) also grows and the topological structure of $F_{\tau }^{*}\left(t\right)$ eventually stabilizes; nevertheless, robust Betti numbers appear at much higher values of τ than with the exponentially decaying links, and the match between them and the physical Betti numbers is much less frequent. Thus, the statistical spread of the connections' lifetimes produced by the tail of the exponential distribution (2) plays an important role in attaining the net complex' stability, i.e., that a certain “synaptic disorder” is required for effective learning (Chowdhury et al., 2018).

Overall, the model suggests that although many details of topological dynamics of flickering complexes may depend on the simplexes' lifetimes and other parameters, several qualitative features, notably the emergent topological stability of F(t) are *universal*, i.e., largely independent from the simplex-recycling mechanisms. In fact, even if the functional connections between place cells are established and pruned randomly, at a rate that matches the statistics (2), the resulting random connectivity graph $G_{r}\left(t\right)$ produces a random clique complex $F_{r}\left(t\right)$ whose Betti numbers converge to the Betti numbers of the environment at the same timescale as the Betti numbers of $F_{\tau }\left(t\right)$ or $F_{\varpi }\left(t\right)$, exhibiting similar pattern of the topological fluctuations. Importantly, in the latter case, the details of these processes are controlled by the physiological parameters, e.g., by the number of active cells and their firing rates (see Figure 6C and Babichev et al., 2018, 2019).

### 3.8. Memory Spaces

In the above discussion, the coactivity complexes were used to describe topological structure of the hippocampal spatial memory frameworks—cognitive maps (Moser et al., 2008; Schmidt and Redish, 2013). However, it is well-known that hippocampus encodes not only spatial but also generic, non-spatial memories (Wood et al., 2000; Ginther et al., 2011; Wixted et al., 2018; Wu et al., 2020), embedding them into broader contexts, placing them in sequence of preceding and succeeding events (Agster et al., 2002; Fortin et al., 2002). In Eichenbaum et al. (1999) it was suggested that the resulting integrated memory structure may be viewed as a *memory space* M that subjects can “mentally explore” or “mentally navigate” (Theves et al., 2020). In other words, it was suggested that individual memory episodes and the spatiotemporal relationships between them may be viewed as “locations” or “regions” that may overlap, contain one another or be otherwise related in a spatial manner (Babichev and Dabaghian, 2018). In particular, the standard spatial inferences that enable spatial cognition and behavior are viewed as particular examples of the memory space navigations (Johnson and Redish, 2007; Hopfield, 2010; Issa and Zhang, 2012; Dabaghian, 2016).

From a physiological perspective, the fact that a memory space associated with a given environment E is encoded by the same place cell population that produces a cognitive map of E, suggests that the corresponding coactivity complex $T_{CA}$ may be used to represent both structures. To gain an insight into this representation, notice that any simplicial complex, in particular $T_{CA}$, defines a finite topological space $A\left(T_{CA}\right)$, endowed with Alexandrov topology: the locations in $A\left(T_{CA}\right)$ correspond to the coactivity simplexes and the topological neighborhoods of a given location represented by a simplex ς are formed by the locations whose simplexes include ς (Alexandroff, 1937; Babichev and Dabaghian, 2018). Since the simplexes of $T_{CA}$ represent combinations of coactive place cells, which, in turn, are believed to represent memory elements, one may view the resulting “topological space of coactivities” $A\left(T_{CA}\right)$ as a representation of the topological memory space encoded by the corresponding cell assembly network, $M=A\left(T_{CA}\right)$. There are three immediate implications of this construction.

*i*. The dynamics of the large-scale topological structure of memory space can be inferred directly from the algebro-topological studies of the corresponding coactivity complexes, since the (singular) homologies of $M\left(T_{CA}\right)$ are identical to the (simplicial) homologies of the coactivity complex $T_{CA}$ (McCord, 1966; Stong, 1966; Babichev et al., 2016a). This implies, e.g., that a memory space that contains a topological map of a given environment emerges over the same learning period *T*_min_ and within the same scope of spiking parameters L as the cognitive map, that it is similarly affected by the brain waves, by the deteriorating synapses, etc., and by the remappings (Babichev and Dabaghian, 2018).

*ii*. It can be shown that neuronal activity representing a trajectory γ traced by the animal in physical space maps continuously into path ℘ navigated in the Alexandrov topology of the memory space $M\left(T_{CA}\right)$. This provides a theoretical base for the intuition of “mental exploration,” allowing to interpret the succession of the place cell activities as a representation of a continuous succession of memory episodes (Samsonovich and McNaughton, 1997; Issa and Zhang, 2012; Buzsáki et al., 2014; Dabaghian, 2016).

*iii*. In neuroscience literature it is recognized that “*space is constructed in the brain rather than perceived, and the hippocampus is central to this construction*,” and yet its meaning remains unclear: “*how can spaceless data enter the hippocampal system and spatial cognitive maps come out*” (O'Keefe and Nadel, 1978; Nadel and Hardt, 2004). The topological model may shed light on these problems, because it allows interpreting spatiality *intrinsically*, as a certain *relational* structure defined on spiking activity (Vickers, 1989; Roeper, 1997; Cohn and Hazarika, 2001), thus providing an ontological foundation for the emergent spatiality of the cognitive map, mentioned in the Introduction.

## 4. Discussion

Extensive studies are dedicated to establishing correlations between parameters of neuronal activity and the characteristics of cognitive phenomena that emerge from this activity (Postle, 2006). The approach discussed above aims at filling the “semantic gap” between these two scales of information processing within a unified framework, based on the conjecture about topological nature of the hippocampal memory organization (Dabaghian et al., 2014; Babichev et al., 2016b; Babichev and Dabaghian, 2018). A formal connection with the realm of simplicial topology is made based on an observation that neuronal computations may be described as operations over spike combinations—which ones are produced over a given period, which ones are detected or transformed into specific outputs, etc. Viewing each particular collection of spikes as an abstract simplex allows representing large volumes of spiking data as abstract simplicial complexes whose topological properties describe the net qualitative information emerging at the neuronal ensemble level. With this approach, the simplicial complex' dynamics may be used as a metaphor for the learning processes, which permits not only phenomenological descriptions at different spatiotemporal scales but also possesses explanatory power, i.e., allows embedding empirical data into qualitative and quantitative schemas for reasoning about cognitive phenomena.

The framework also allows describing the flow of information in transient networks, which significantly expands the scope of the modeled phenomena. The net structure of this information is represented by flickering coactivity complexes that exhibit topological dynamics at three complementary timescales. The fastest timescale corresponds to rapid recycling of the local connections, which represents the flow of the ongoing, temporary information—the *short-term memory* (Hebb, 1949; Cowan, 2008). The net topological dynamics, described by the time-dependent invariants, e.g., Betti numbers, unfolds at a timescale that is by about an order of magnitude slower than the simplex-level fluctuations. Physiologically, this “operational” timescale corresponds to the *intermediate-term memory* (Eichenbaum et al., 1994; Kesner and Hunsaker, 2010). Lastly, the topological variations occur over a robust base that marks persistent, qualitative characteristics that marks the *long-term memory*. Such stratification indicates functional importance of the complementary learning systems for processing information at different levels of spatiotemporal granularity (O'Reilly and McClelland, 1994; McClelland et al., 1995; Fusi et al., 2005).

The model reveals complex interactions between these dynamics; for example, for sufficiently slow transience rates, the fluctuations of the topological shapes encoded by the network freeze out, i.e., the simulated cognitive map can acquire topological stability. Physiologically, this implies that if the cell assemblies rewire sufficiently slowly, then the net map encoded by the corresponding network may retain its structure despite the recycling connections in its neuronal substrate. In other words, the model suggests that synaptic and structural plasticity, which are ultimately responsible for the network's ability to incorporate new information (McHugh and Tonegawa, 2009; Leuner et al., 2010; Schaefers et al., 2010), do not necessarily compromise the qualitative information represented by the system. Rather, renewing connections allow correcting errors, e.g., removing spurious topological defects that may have appeared by an accident. As a result, a network capable of recycling information demonstrates better learning capacity, suggesting that both learning and forgetting components are necessary for physiological learning (Dupret et al., 2010; Kuhl et al., 2010; Murre et al., 2013). The model also suggests that memory deterioration caused by an overly rapid decay of the network's connections may be compensated by increasing neuronal activity, e.g., by boosting the neuronal firing rates (Babichev et al., 2018) or by increasing the “off-line,” endogenous activity of the hippocampal network that can occur in wake or in sleep states (Ji and Wilson, 2007; Karlsson and Frank, 2009; Dragoi and Tonegawa, 2011, 2013). In certain contexts, such *replays* can be viewed as manifestations of the animal's “mental explorations” of its cognitive map (Foster and Wilson, 2006; Johnson and Redish, 2007; Hopfield, 2010; Issa and Zhang, 2012; Dabaghian, 2016), which are believed to help learning and memory consolidation (Girardeau et al., 2010; Roux et al., 2017). Indeed, the model shows that frequent place cell replays significantly reduce the structural fluctuations in the cognitive map, thus helping to separate the fast and the slow timescales and to extract stable, qualitative representation of the external world (Babichev et al., 2019).

## Data Availability Statement

The original contributions presented in the study are included in the article/supplementary material, further inquiries can be directed to the corresponding author/s.

## Author Contributions

YD conceived and wrote this manuscript.

## Conflict of Interest

The author declares that the research was conducted in the absence of any commercial or financial relationships that could be construed as a potential conflict of interest.

## References

[B1] Agarwal G. Stevenson I. Berényi A. Mizuseki K. Buzsáki G. Sommer F. (2014). Spatially distributed local fields in the hippocampus encode rat position. Science 344, 626–630. 10.1126/science.1250444 24812401 PMC4909490

[B2] Agster K. Fortin N. Eichenbaum H. (2002). The hippocampus and disambiguation of overlapping sequences. J. Neurosci. 22, 5760–5768. 10.1523/JNEUROSCI.22-13-05760.2002 12097529 PMC4053169

[B3] Albert R. Barabási A.-L. (2002). Statistical mechanics of complex networks. Rev. Mod. Phys. 74, 47–97. 10.1103/RevModPhys.74.47

[B4] Alexandroff P. (1928). Untersuchungen über Gestalt und Lage abgeschlossener Mengen beliebiger Dimension. Ann. Math. 30, 101–187. 10.2307/1968272

[B5] Alexandroff P. (1937). Diskrete Räume. Rec. Math. 2, 501–518.

[B6] Alexandrov P. (1965). Elementary Concepts of Topology. New York, NY: F. Ungar Pub. Co.

[B7] Alvernhe A. Sargolini F. Poucet B. (2012). Rats build and update topological representations through exploration. Anim. Cogn. 15, 359–368. 10.1007/s10071-011-0460-z 21915695

[B8] Ang C. Carlson G. Coulter D. (2005). Hippocampal CA1 circuitry dynamically gates direct cortical inputs preferentially at theta frequencies. J. Neurosci. 25, 9567–9580. 10.1523/JNEUROSCI.2992-05.2005 16237162 PMC2048747

[B9] Arai M. Brandt V. Dabaghian Y. (2014). The effects of theta precession on spatial learning and simplicial complex dynamics in a topological model of the hippocampal spatial map. PLoS Comput. Biol. 10:e1003651. 10.1371/journal.pcbi.1003651 24945927 PMC4063672

[B10] Babichev A. Cheng S. Dabaghian Y. (2016b). Topological schemas of cognitive maps and spatial learning. Front. Comput. Neurosci. 10:18. 10.3389/fncom.2016.00018 27014045 PMC4781836

[B11] Babichev A. Dabaghian Y. (2017a). Persistent memories in transient networks. Springer Proc. Phys. 191, 179–188 10.1007/978-3-319-47810-4_14

[B12] Babichev A. Dabaghian Y. (2017b). Transient cell assembly networks encode stable spatial memories. Sci. Rep. 7:3959. 10.1038/s41598-017-03423-3 28638123 PMC5479874

[B13] Babichev A. Dabaghian Y. (2018). Topological schemas of memory spaces. Front. Comput. Neurosci. 12:27. 10.3389/fncom.2018.00027 29740306 PMC5928258

[B14] Babichev A. Ji D. Memoli F. Dabaghian Y. (2016a). A topological model of the hippocampal cell assembly network. Front. Comput. Neurosci. 10:50. 10.3389/fncom.2016.00050 27313527 PMC4889593

[B15] Babichev A. Morozov D. Dabaghian Y. (2018). Robust spatial memory maps encoded by networks with transient connections. PLoS Comput. Biol. 14:e1006433. 10.1371/journal.pcbi.1006433 30226836 PMC6161922

[B16] Babichev A. Morozov D. Dabaghian Y. (2019). Replays of spatial memories suppress topological fluctuations in cognitive map. Netw. Neurosci. 3, 707–724. 10.1162/netn_a_00076 31410375 PMC6663216

[B17] Barabási A.-L Albert R. (1999). Emergence of scaling in random networks. Science 286, 509–512. 10.1126/science.286.5439.509 10521342

[B18] Barbieri R. Frank L. Nguyen D. Quirk M. Solo V. Wilson M.. (2004). Dynamic analyses of information encoding in neural ensembles. Neural Comput. 16, 277–307. 10.1162/089976604322742038 15006097

[B19] Barbour B. Brunel N. Hakim V. Nadal J.-P. (2007). What can we learn from synaptic weight distributions? Trends Neurosci. 30, 622–629. 10.1016/j.tins.2007.09.005 17983670

[B20] Basso E. Arai M. Dabaghian Y. (2016). Gamma synchronization influences map formation time in a topological model of spatial learning. PLoS Comput. Biol. 12:e1005114. 10.1371/journal.pcbi.1005114 27636199 PMC5026372

[B21] Battiston F. Cencetti G. Iacopini I. Latora V. Lucash M. Patania A. (2020). Networks beyond pairwise interactions: structure and dynamics. Phys. Rep. 874, 1–92. 10.1016/j.physrep.2020.05.004

[B22] Bellmund J. de Cothi W. Ruiter T. Nau M. Barry C. Doeller C. (2020). Deforming the metric of cognitive maps distorts memory. Nat. Hum. Behav. 4, 177–188. 10.1038/s41562-019-0767-3 31740749 PMC7619669

[B23] Bennett S. Kirby A. Finnerty G. (2018). Rewiring the connectome: evidence and effects. Neurosci. Biobehav. Rev. 88, 51–62. 10.1016/j.neubiorev.2018.03.001 29540321 PMC5903872

[B24] Bi G. Poo M. (2001). Synaptic modification by correlated activity: Hebb's postulate revisited. Annu. Rev. Neurosci. 24, 139–166. 10.1146/annurev.neuro.24.1.139 11283308

[B25] Billeh Y. Schaub M. Anastassiou C. Barahona M. Koch C. (2014). Revealing cell assemblies at multiple levels of granularity. J. Neurosci. Methods 236, 92–106. 10.1016/j.jneumeth.2014.08.011 25169050

[B26] Bonifazi P. Goldin M. Picardo M. Jorquera I. Cattani A. Bianconi G.. (2009). GABAergic hub neurons orchestrate synchrony in developing hippocampal networks. Science 326, 1419–1424. 10.1126/science.1175509 19965761

[B27] Brette R. (2012). Computing with neural synchrony. PLoS Comput. Biol. 8:e1002561. 10.1371/journal.pcbi.1002561 22719243 PMC3375225

[B28] Brown E. Frank L. Tang D. Quirk M. Wilson M. (1998). A statistical paradigm for neural spike train decoding applied to position prediction from ensemble firing patterns of rat hippocampal place cells. J. Neurosci. 18, 7411–7425. 10.1523/JNEUROSCI.18-18-07411.1998 9736661 PMC6793233

[B29] Brown M. Farley R. Lorek E. (2007). Remembrance of places you passed: social spatial working memory in rats. J. Exp. Psychol. Anim. Behav. Process. 33, 213–224. 10.1037/0097-7403.33.3.213 17620022

[B30] Brunel N. Hakim V. Isope P. Nadal J.-P. Barbour B. (2004). Optimal information storage and the distribution of synaptic weights: perceptron versus purkinje cell. Neuron 43, 745–757. 10.1016/S0896-6273(04)00528-8 15339654

[B31] Burgess N. O'Keefe J. (1996). Cognitive graphs, resistive grids, and the hippocampal representation of space. J. Gen. Physiol. 107, 659–662. 10.1085/jgp.107.6.659 8783069 PMC2219393

[B32] Buzsáki G. (2002). Theta oscillations in the hippocampus. Neuron 33, 325–340. 10.1016/S0896-6273(02)00586-X 11832222

[B33] Buzsáki G. (2005). Theta rhythm of navigation: link between path integration and landmark navigation, episodic and semantic memory. Hippocampus 15, 827–840. 10.1002/hipo.20113 16149082

[B34] Buzsaki G. (2010). Neural syntax: cell assemblies, synapsembles, and readers. Neuron 68, 362–385. 10.1016/j.neuron.2010.09.023 21040841 PMC3005627

[B35] Buzsáki G. Draguhn A. (2004). Neuronal oscillations in cortical networks. Science 304, 1926–1929. 10.1126/science.1099745 15218136

[B36] Buzsáki G. Mizuseki K. (2014). The log-dynamic brain: how skewed distributions affect network operations. Nat. Rev. Neurosci. 15, 264–278. 10.1038/nrn3687 24569488 PMC4051294

[B37] Buzsáki G. Peyrache A. Kubie J. (2014). Emergence of cognition from action. Cold Spring Harb. Symp. Quant. Biol. 79, 41–50. 10.1101/sqb.2014.79.024679 25752314 PMC4895837

[B38] Buzsáki G. Wang X. (2012). Mechanisms of gamma oscillations. Annu. Rev. Neurosci. 35, 203–225. 10.1146/annurev-neuro-062111-150444 22443509 PMC4049541

[B39] Cacucci F. Yi M. Wills T. Chapman P. O'Keefe J. (2008). Place cell firing correlates with memory deficits and amyloid plaque burden in Tg2576 Alzheimer mouse model. Proc. Natl. Acad. Sci. U.S.A. 105, 7863–7868. 10.1073/pnas.0802908105 18505838 PMC2396558

[B40] Carlsson G. (2009). Topology and data. Bull. Amer. Math. Soc. 46, 255–308. 10.1090/S0273-0979-09-01249-X

[B41] Carlsson G. Silva Vd. (2010). Zigzag persistence. Found. Comput. Math. 10, 367–405. 10.1007/s10208-010-9066-0

[B42] Carlsson G. Silva Vd. Morozov D. (2009). Zigzag persistent homology and real-valued functions, in Proceedings of the 25th Annual Symposium on Computational Geometry (Aarhus: ACM), 247–256. 10.1145/1542362.1542408

[B43] Čech E. (1932). Théorie générale de l'homologie dans un espace quelconque. Fundam. Math. 19, 149–183. 10.4064/fm-19-1-149-183

[B44] Chen Z. Kloosterman F. Brown E. Wilson M. (2012). Uncovering spatial topology represented by rat hippocampal population neuronal codes. J. Comput. Neurosci. 33, 227–255. 10.1007/s10827-012-0384-x 22307459 PMC3974406

[B45] Chowdhury S. Dai B. Memoli F. (2018). The importance of forgetting: limiting memory improves recovery of topological characteristics from neural data. PLoS ONE 13:e0202561. 10.1371/journal.pone.0202561 30180172 PMC6122934

[B46] Clayton N. Bussey T. Dickinson A. (2003). Can animals recall the past and plan for the future? Nat. Rev. Neurosci. 4, 685–691. 10.1038/nrn1180 12894243

[B47] Cohen R. Rezai-Zadeh K. Weitz T. Rentsendorj A. Gate D. Spivak I.. (2013). A transgenic Alzheimer rat with plaques, tau pathology, behavioral impairment, oligomeric Aβ, and Frank neuronal loss. J. Neurosci. 33, 6245–6256. 10.1523/JNEUROSCI.3672-12.2013 23575824 PMC3720142

[B48] Cohn A. G. Hazarika S. M. (2001). Qualitative spatial representation and reasoning: an overview. Fundam. Inf. 46, 1–29. 10.5555/1219982.1219984

[B49] Colgin L. Denninger T. Fyhn M. Hafting T. Bonnevie T. Jensen O.. (2009). Frequency of gamma oscillations routes flow of information in the hippocampus. Nature 462, 353–357. 10.1038/nature08573 19924214

[B50] Colgin L. Moser E. (2010). Gamma oscillations in the hippocampus. Physiology 25, 319–329. 10.1152/physiol.00021.2010 20940437

[B51] Cowan N. (2008). What are the differences between long-term, short-term, and working memory? Prog. Brain Res. 169, 323–338. 10.1016/S0079-6123(07)00020-9 18394484 PMC2657600

[B52] Curto C. Itskov V. (2008). Cell groups reveal structure of stimulus space. PLoS Comput. Biol. 4:e1000205. 10.1371/journal.pcbi.1000205 18974826 PMC2565599

[B53] Dabaghian Y. (2016). Maintaining consistency of spatial information in the hippocampal network: a combinatorial geometry model. Neural Comput. 28, 1051–1071. 10.1162/NECO_a_00840 27137840 PMC6223651

[B54] Dabaghian Y. (2019). Through synapses to spatial memory maps: a topological model. Sci. Rep. 9:572. 10.1038/s41598-018-36807-0 30679520 PMC6345962

[B55] Dabaghian Y. Brandt V. Frank L. (2014). Reconceiving the hippocampal map as a topological template. eLife 3:e03476. 10.7554/eLife.03476.009 25141375 PMC4161971

[B56] Dabaghian Y. Memoli F. Frank L. Carlsson G. (2012). A topological paradigm for hippocampal spatial map formation using persistent homology. PLoS Comput. Biol. 8:e1002581. 10.1371/journal.pcbi.1002581 22912564 PMC3415417

[B57] De Silva V. Ghrist R. (2007). Coverage in sensor networks via persistent homology. Algebr. Geometr. Topol. 7, 339–358. 10.2140/agt.2007.7.339

[B58] Dilgen J. Tompa T. Saggu S. Naselaris T. Lavin A. (2013). Optogenetically evoked gamma oscillations are disturbed by cocaine administration. Front. Cell Neurosci. 7:213. 10.3389/fncel.2013.00213 24376397 PMC3841795

[B59] Donato I. Gori M. Pettini M. Petri G. De Nigris S. Franzosi R.. (2016). Persistent homology analysis of phase transitions. Phys. Rev. E 93:052138. 10.1103/PhysRevE.93.052138 27300860

[B60] Dragoi G. Tonegawa S. (2011). Preplay of future place cell sequences by hippocampal cellular assemblies. Nature 469, 397–401. 10.1038/nature09633 21179088 PMC3104398

[B61] Dragoi G. Tonegawa S. (2013). Distinct preplay of multiple novel spatial experiences in the rat. Proc. Natl. Acad. Sci. U.S.A. 110, 9100–9105. 10.1073/pnas.1306031110 23671088 PMC3670374

[B62] Dupret D. Pleydell-Bouverie B. Csicsvari J. (2010). Rate remapping: when the code goes beyond space. Neuron 68, 1015–1016. 10.1016/j.neuron.2010.12.011 21172603

[B63] Eckert M. Abraham W. (2010). Physiological effects of enriched environment exposure and LTP induction in the hippocampus *in vivo* do not transfer faithfully to *in vitro* slices. Learn. Mem. 17, 480–484. 10.1101/lm.1822610 20861169 PMC2948890

[B64] Edelsbrunner H. Harer J. (2010). Computational topology: an introduction. Am. Math. Soc. (Cambridge, NY: Cambridge University Press), 241 10.1090/mbk/069

[B65] Edelsbrunner H. Letscher D. Zomorodian A. (2002). Topological persistence and simplification. Discrete Computat. Geom. 28, 511—533. 10.1007/s00454-002-2885-2

[B66] Eichenbaum H. Dudchenko P. Wood E. Shapiro M. Tanila H. (1999). The hippocampus, memory, and place cells: is it spatial memory or a memory space? Neuron 23, 209–226. 10.1016/S0896-6273(00)80773-4 10399928

[B67] Eichenbaum H. Otto T. Cohen N. (1994). Two functional components of the hippocampal memory system. Behav. Brain Sci. 17, 449–472. 10.1017/S0140525X00035391

[B68] Fenton A. Kao H. Neymotin S. Olypher A. Vayntrub Y. Lytton W.. (2008). Unmasking the CA1 ensemble place code by exposures to small and large environments: more place cells and multiple, irregularly arranged, and expanded place fields in the larger space. J. Neurosci. 28, 11250–11262. 10.1523/JNEUROSCI.2862-08.2008 18971467 PMC2695947

[B69] Fenton A. Muller R. (1998). Place cell discharge is extremely variable during individual passes of the rat through the firing field. Proc. Natl. Acad. Sci. U.S.A. 95, 3182–3187. 10.1073/pnas.95.6.3182 9501237 PMC19716

[B70] Fortin N. Agster K. Eichenbaum H. (2002). Critical role of the hippocampus in memory for sequences of events. Nat. Neurosci. 5, 458–462. 10.1038/nn834 11976705 PMC4053170

[B71] Foster D. Wilson M. (2006). Reverse replay of behavioural sequences in hippocampal place cells during the awake state. Nature 440, 680–683. 10.1038/nature04587 16474382

[B72] Fusi S. Drew P. Abbott L. (2005). Cascade models of synaptically stored memories. Neuron 45, 599–611. 10.1016/j.neuron.2005.02.001 15721245

[B73] Geisler C. Diba K. Pastalkova E. Mizuseki K. Royer S. Buzsáki G. (2010). Temporal delays among place cells determine the frequency of population theta oscillations in the hippocampus. Proc. Natl. Acad. Sci. U.S.A. 107, 7957–7962. 10.1073/pnas.0912478107 20375279 PMC2867922

[B74] Ghrist R. (2008). Barcodes: the persistent topology of data. Bull. Am. Math. Soc. 45, 61–75. 10.1090/S0273-0979-07-01191-3

[B75] Ginther M. Walsh D. Ramus S. (2011). Hippocampal neurons encode different episodes in an overlapping sequence of odors task. J. Neurosci. 31, 2706–2711. 10.1523/JNEUROSCI.3413-10.2011 21325539 PMC3047458

[B76] Girardeau G. Benchenane K. Wiener S. Buzsaki G. Zugaro M. (2010). Selective suppression of hippocampal ripples impairs spatial memory. Nat. Neurosci. 12, 1222–1223. 10.1038/nn.2384 19749750

[B77] Goldman-Rakic P. (1995). Cellular basis of working memory. Neuron 14, 477–485. 10.1016/0896-6273(95)90304-6 7695894

[B78] Gothard K. Skaggs W. McNaughton B. (1996). Dynamics of mismatch correction in the hippocampal ensemble code for space: interaction between path integration and environmental cues. J. Neurosci. 16, 8027–8040. 10.1523/JNEUROSCI.16-24-08027.1996 8987829 PMC6579211

[B79] Guger C. Gener T. Pennartz C. Brotons-Mas J. Edlinger G. Badia S. B. I.. (2011). Real-time position reconstruction with hippocampal place cells. Front. Neurosci. 5:85. 10.3389/fnins.2011.00085 21808603 PMC3129134

[B80] Harris K. (2005). Neural signatures of cell assembly organization. Nat. Rev. Neurosci. 6, 399–407. 10.1038/nrn1669 15861182

[B81] Harris K. Henze D. Hirase H. Leinekugel X. Dragoi G. Czurkó A.. (2002). Spike train dynamics predicts theta-related phase precession in hippocampal pyramidal cells. Nature 417, 738–741. 10.1038/nature00808 12066184

[B82] Harris K. D. Csicsvari J. Hirase H. Dragoi G. Buzsaki G. (2003). Organization of cell assemblies in the hippocampus. Nature 424, 552–556. 10.1038/nature01834 12891358

[B83] Hasselmo M. Bodelon C. Wyble B. (2002). A proposed function for hippocampal theta rhythm: separate phases of encoding and retrieval enhance reversal of prior learning. Neural Comput. 14, 793–817. 10.1162/089976602317318965 11936962

[B84] Hatcher A. (2002). Algebraic Topology. Cambridge: Cambridge University Press.

[B85] Hebb D. (1949). The Organization of Behavior; A Neuropsychological Theory. New York, NY: Wiley.

[B86] Hiratani N. Fukai T. (2014). Interplay between short- and long-term plasticity in cell-assembly formation. PLoS ONE 9:e101535. 10.1371/journal.pone.0101535 25007209 PMC4090127

[B87] Hoffman K. Babichev A. Dabaghian Y. (2016). A model of topological mapping of space in bat hippocampus. Hippocampus 26, 1345–1353. 10.1002/hipo.22610 27312850

[B88] Hopfield J. (2010). Neurodynamics of mental exploration. Proc. Natl. Acad. Sci. U.S.A. 107, 1648–1653. 10.1073/pnas.0913991107 20080534 PMC2824418

[B89] Huhn Z. Orbán G. Érdi P. Lengyel M. (2005). Theta oscillation-coupled dendritic spiking integrates inputs on a long time scale. Hippocampus 15, 950–962. 10.1002/hipo.20112 16108010

[B90] Huxter J. Senior T. Allen K. Csicsvari J. (2008). Theta phase-specific codes for two-dimensional position, trajectory and heading in the hippocampus. Nat. Neurosci. 11, 587–594. 10.1038/nn.2106 18425124

[B91] Issa J. Zhang K. (2012). Universal conditions for exact path integration in neural systems. Proc. Natl. Acad. Sci. U.S.A. 109, 6716–6720. 10.1073/pnas.1119880109 22493275 PMC3340063

[B92] Jackson J. Redish A. (2007). Network dynamics of hippocampal cell-assemblies resemble multiple spatial maps within single tasks. Hippocampus 17, 1209–1229. 10.1002/hipo.20359 17764083

[B93] Jensen O. Lisman J. (1996). Hippocampal CA3 region predicts memory sequences: accounting for the phase precession of place cells. Learn. Mem. 3, 279–287. 10.1101/lm.3.2-3.279 10456097

[B94] Jensen O. Lisman J. (2000). Position reconstruction from an ensemble of hippocampal place cells: contribution of theta phase coding. J. Neurophysiol. 83, 2602–2609. 10.1152/jn.2000.83.5.2602 10805660

[B95] Jezek K. Henriksen E. Treves A. Moser E. Moser M.-B. (2011). Theta-paced flickering between place-cell maps in the hippocampus. Nature 478, 246–249. 10.1038/nature10439 21964339

[B96] Ji D. Wilson M. (2007). Coordinated memory replay in the visual cortex and hippocampus during sleep. Nat. Neurosci. 10, 100–107. 10.1038/nn1825 17173043

[B97] Jia X. Kohn A. (2011). Gamma rhythms in the brain. PLoS Biol. 9:e1001045. 10.1371/journal.pbio.1001045 21556334 PMC3084194

[B98] Johnson A. Redish A. (2007). Neural ensembles in CA3 transiently encode paths forward of the animal at a decision point. J. Neurosci. 27, 12176–12189. 10.1523/JNEUROSCI.3761-07.2007 17989284 PMC6673267

[B99] Jonsson J. (2008). Simplicial Complexes of Graphs. New York, NY: Springer.

[B100] Kang L. Xu B. Morozov D. (2020). State space discovery in spatial representation circuits with persistent cohomology. bioRxiv 2020.2010.2006.328773 10.1101/2020.10.06.328773

[B101] Karlsson M. Frank L. (2009). Awake replay of remote experiences in the hippocampus. Nat. Neurosci. 12, 913–918. 10.1038/nn.2344 19525943 PMC2750914

[B102] Katz Y. Kath W. Spruston N. Hasselmo M. (2007). Coincidence detection of place and temporal context in a network model of spiking hippocampal neurons. PLoS Comput. Biol. 3:e234. 10.1371/journal.pcbi.0030234 18085816 PMC2134961

[B103] Kesner R. Hunsaker M. (2010). The temporal attributes of episodic memory. Behav. Brain Res. 215, 299–309. 10.1016/j.bbr.2009.12.029 20036694

[B104] König P. Engel A. Singer W. (1996). Integrator or coincidence detector? The role of the cortical neuron revisited. Trends Neurosci. 19, 130–137. 10.1016/S0166-2236(96)80019-1 8658595

[B105] Kuhl B. Shah A. DuBrow S. Wagner A. (2010). Resistance to forgetting associated with hippocampus-mediated reactivation during new learning. Nat. Neurosci. 13, 501–506. 10.1038/nn.2498 20190745 PMC2847013

[B106] Lee I. Yoganarasimha D. Rao G. Knierim J. (2004). Comparison of population coherence of place cells in hippocampal subfields CA1 and CA3. Nature 430, 456–459. 10.1038/nature02739 15229614

[B107] Leutgeb J. Leutgeb S. Treves A. Meyer R. Barnes C. McNaughton B.. (2005). Progressive transformation of hippocampal neuronal representations in “morphed” environments. Neuron 48, 345–358. 10.1016/j.neuron.2005.09.007 16242413

[B108] Li X. Ouyang G. Usami A. Ikegaya Y. Sik A. (2010). Scale-free topology of the CA3 hippocampal network: a novel method to analyze functional neuronal assemblies. Biophys. J. 98, 1733–1741. 10.1016/j.bpj.2010.01.013 20441736 PMC2862192

[B109] Lisman J. (2005). The theta/gamma discrete phase code occuring during the hippocampal phase precession may be a more general brain coding scheme. Hippocampus 15, 913–922. 10.1002/hipo.20121 16161035

[B110] Lisman J. Idiart M. (1995). Storage of 7 ± 2 short-term memories in oscillatory subcycles. Science 267, 1512–1515. 10.1126/science.7878473 7878473

[B111] London M. Häusser M. (2005). Dendritic computation. Ann. Rev. Neurosci. 28, 503–532. 10.1146/annurev.neuro.28.061604.135703 16033324

[B112] Lu C. Hamilton J. Powell A. Toescu E. Vreugdenhil M. (2011). Effect of ageing on CA3 interneuron sAHP and γ oscillations is activity-dependent. Neurobiol. Aging 32, 956–965. 10.1016/j.neurobiolaging.2009.05.006 19523715

[B113] Lum P. Singh G. Lehman A. Ishkanov T. Vejdemo-Johansson M. Alagappan M.. (2013). Extracting insights from the shape of complex data using topology Sci. Rep. 3:1236. 10.1038/srep01236 23393618 PMC3566620

[B114] Lundqvist M. Herman P. Lansner A. (2011). Theta and gamma power increases and alpha/beta power decreases with memory load in an attractor network model. J. Cogn. Neurosci. 23, 3008–3020. 10.1162/jocn_a_00029 21452933

[B115] Magee J. (2000). Dendritic integration of excitatory synaptic input. Nat. Rev. Neurosci. 1, 181–190. 10.1038/35044552 11257906

[B116] Matthews D. Simson P. Best P. (1996). Ethanol alters spatial processing of hippocampal place cells: a mechanism for impaired navigation when intoxicated. Alcohol Clin. Exp. Res. 20, 404–407. 10.1111/j.1530-0277.1996.tb01660.x 8730237

[B117] Maurer A. Cowen S. Burke S. Barnes C. McNaughton B. (2006). Organization of hippocampal cell assemblies based on theta phase precession. Hippocampus 16, 785–794. 10.1002/hipo.20202 16921501

[B118] McClelland J. McNaughton B. O'Reilly R. (1995). Why there are complementary learning systems in the hippocampus and neocortex: insights from the successes and failures of connectionist models of learning and memory. Psychol. Rev. 102, 419–457 10.1037/0033-295X.102.3.419 7624455

[B119] McCord M. C. (1966). Singular homology groups and homotopy groups of finite topological spaces. Duke Math. J. 33, 465–474. 10.1215/S0012-7094-66-03352-7

[B120] McCracken C. Grace A. (2013). Persistent cocaine-induced reversal learning deficits are associated with altered limbic cortico-striatal local field potential synchronization. J. Neurosci. 33, 17469–17482. 10.1523/JNEUROSCI.1440-13.2013 24174680 PMC3812511

[B121] McHugh T. Tonegawa S. (2009). CA3 NMDA receptors are required for the rapid formation of a salient contextual representation. Hippocampus 19, 1153–1158. 10.1002/hipo.20684 19650121 PMC2788054

[B122] McHugh T. Tonegawa S. Leuner B. Gould E. (2010). Structural plasticity and hippocampal function. Annu. Rev. Psychol. 61, 111–140. 10.1146/annurev.psych.093008.100359 19575621 PMC3012424

[B123] Meck W. Church R. Olton D. (2013). Hippocampus, time, and memory. Behav. Neurosci. 127, 655–668. 10.1037/a0034188 24128355

[B124] Mizuseki K. Sirota A. Pastalkova E. Buzsáki G. (2009). Theta oscillations provide temporal windows for local circuit computation in the entorhinal-hippocampal loop. Neuron, 64, 267–280. 10.1016/j.neuron.2009.08.037 19874793 PMC2771122

[B125] Moretti D. Fracassi C. Pievani M. Geroldi C. Binetti G. Zanetti O.. (2009). Increase of θ/γ ratio is associated with memory impairment. Clin. Neurophysiol. 120, 295–303. 10.1016/j.clinph.2008.11.012 19121602

[B126] Moser E. I. Kropff E. Moser M-B. (2008). Place cells, grid cells, and the brain's spatial representation system. Annu. Rev. Neurosci. 31, 69–89. 10.1146/annurev.neuro.31.061307.090723 18284371

[B127] Muller R. Stead M. Pach J. (1996). The hippocampus as a cognitive graph. J. Gen. Physiol. 107, 663–694. 10.1085/jgp.107.6.663 8783070 PMC2219396

[B128] Murre J. Chessa A. Meeter M. (2013). A mathematical model of forgetting and amnesia. Front. Psychol. 4:76. 10.3389/fpsyg.2013.00076 23450438 PMC3584298

[B129] Nadel L. Hardt O. (2004). The spatial brain. Neuropsychology 18, 473–476. 10.1037/0894-4105.18.3.473 15291725

[B130] Neves G. Cooke S. Bliss T. (2008). Synaptic plasticity, memory and the hippocampus: a neural network approach to causality. Nat. Rev. Neurosci. 9, 65–75. 10.1038/nrn2303 18094707

[B131] Nikoli D. Fries P. Singer W. (2013). Gamma oscillations: precise temporal coordination without a metronome. Trends Cogn. Sci. 17, 54–55. 10.1016/j.tics.2012.12.003 23287106

[B132] Nithianantharajah J. Hannan A. (2006). Enriched environments, experience-dependent plasticity and disorders of the nervous system. Nat. Rev. Neurosci. 7, 697–709. 10.1038/nrn1970 16924259

[B133] O'Keefe J. Nadel L. (1978). The Hippocampus as a Cognitive Map. New York, NY: Clarendon Press; Oxford University Press.

[B134] O'Neill J. Senior T. Allen K. Huxter J. Csicsvari J. (2008). Reactivation of experience-dependent cell assembly patterns in the hippocampus. Nat. Neurosci. 11, 209–215. 10.1038/nn2037 18193040

[B135] O'Reilly R. McClelland J. (1994). Hippocampal conjunctive encoding, storage, and recall: avoiding a trade-off. Hippocampus 4, 661–682. 10.1002/hipo.450040605 7704110

[B136] Perea J. (2019). Topological time series analysis. Notices Am. Math. Soc. 66, 686–693. 10.1090/noti1869

[B137] Place R. Nitz D. (2020). Cognitive maps: distortions of the hippocampal space map define neighborhoods. Curr. Biol. 30, R340–R342. 10.1016/j.cub.2020.02.085 32315629

[B138] Postle B. (2006). Working memory as an emergent property of the mind and brain. Neuroscience 139, 23–38. 10.1016/j.neuroscience.2005.06.005 16324795 PMC1428794

[B139] Pouget A. Dayan P. Zemel R. (2000). Information processing with population codes. Nat. Rev. Neurosci. 1, 125–132. 10.1038/35039062 11252775

[B140] Ratté S. Lankarany M. Rho Y.-A. Patterson A. Prescott S. (2015). Subthreshold membrane currents confer distinct tuning properties that enable neurons to encode the integral or derivative of their input. Front. Cell Neurosci. 8:452. 10.3389/fncel.2014.00452 25620913 PMC4288132

[B141] Robbe D. Buzsáki G. (2009). Alteration of theta timescale dynamics of hippocampal place cells by a cannabinoid is associated with memory impairment. J. Neurosci. 29, 12597–12605. 10.1523/JNEUROSCI.2407-09.2009 19812334 PMC2799373

[B142] Roeper P. (1997). Region-based topology. J. Philos. Logic 26, 251–309. 10.1023/A:1017904631349

[B143] Roux L. Hu B. Eichler R. Stark E. Buzsaki G. (2017). Sharp wave ripples during learning stabilize the hippocampal spatial map. Nat. Neurosci. 20, 845–853. 10.1038/nn.4543 28394323 PMC5446786

[B144] Samsonovich A. McNaughton B. L. (1997). Path integration and cognitive mapping in a continuous attractor neural network model. J Neurosci. 17, 5900–5920. 10.1523/JNEUROSCI.17-15-05900.1997 9221787 PMC6573219

[B145] Schaefers A. Grafen K. Teuchert-Noodt G. Winter Y. (2010). Synaptic remodeling in the dentate gyrus, CA3, CA1, subiculum, and entorhinal cortex of mice: effects of deprived rearing and voluntary running. Neural Plast. 2010:11. 10.1155/2010/870573 20508828 PMC2876250

[B146] Schmidt B. Redish A. (2013). Neuroscience: navigation with a cognitive map. Nature 497, 42–43. 10.1038/nature12095 23594740 PMC5029279

[B147] Selkoe D. (2002). Alzheimer's disease is a synaptic failure. Science 298, 789–791. 10.1126/science.1074069 12399581

[B148] Shapiro M. (2001). Plasticity, hippocampal place cells, and cognitive maps. Arch. Neurol. 58, 874–881. 10.1001/archneur.58.6.874 11405801

[B149] Shepherd G. (2004). The Synaptic Organization of the Brain. 5th Edn. Oxford; New York, NY: Oxford University Press.

[B150] Skaggs W. McNaughton B. Wilson M. Barnes C. (1996). Theta phase precession in hippocampal neuronal populations and the compression of temporal sequences. Hippocampus 6, 149–172. 10.1002/(SICI)1098-1063(1996)6:2<149::AID-HIPO6>3.0.CO;2-K 8797016

[B151] Spruston N. (2008). Pyramidal neurons: dendritic structure and synaptic integration. Nat. Rev. Neurosci. 9, 206–221. 10.1038/nrn2286 18270515

[B152] Stong R. E. (1966). Finite topological spaces. Trans. Am. Math. Soc. 123, 325–340. 10.1090/S0002-9947-1966-0195042-2

[B153] Tancer M. (2013). Intersection patterns of convex sets via simplicial complexes: a survey, in Thirty Essays on Geometric Graph Theory, ed Pach J. (New York, NY: Springer), 521–540. 10.1007/978-1-4614-0110-0_28

[B154] Theunissen F. Miller J. (1995). Temporal encoding in nervous systems: a rigorous definition. J. Comput. Neurosci. 2, 149–162. 10.1007/BF00961885 8521284

[B155] Theves S. Fernández G. Doeller C. (2020). The hippocampus maps concept space, not feature space. J. Neurosci. 40, 7318–7325. 10.1523/JNEUROSCI.0494-20.2020 32826311 PMC7534914

[B156] Thompson L. Best P. (1990). Long-term stability of the place-field activity of single units recorded from the dorsal hippocampus of freely behaving rats. Brain Res. 509, 299–308. 10.1016/0006-8993(90)90555-P 2322825

[B157] Toth M. Melentijevic I. Shah L. Bhatia A. Lu K. Talwar A.. (2012). Neurite sprouting and synapse deterioration in the aging *Caenorhabditis elegans* nervous system. J. Neurosci. 32, 8778–8790. 10.1523/JNEUROSCI.1494-11.2012 22745480 PMC3427745

[B158] Touretzky D. Weisman W. Fuhs M. Skaggs W. Fenton A. Muller R. (2005). Deforming the hippocampal map. Hippocampus 15, 41–55. 10.1002/hipo.20029 15390166

[B159] Trimper J. Stefanescu R. Manns J. (2014). Recognition memory and θ−γ interactions in the hippocampus. Hippocampus 24, 341–353. 10.1002/hipo.22228 24227610 PMC4004109

[B160] Ulanovsky N. Moss C. (2007). Hippocampal cellular and network activity in freely moving echolocating bats. Nat. Neurosci. 10, 224–233. 10.1038/nn1829 17220886

[B161] van Vugt M. Schulze-Bonhage A. Litt B. Brandt A. Kahana M. (2010). Hippocampal gamma oscillations increase with memory load. J. Neurosci. 30, 2694–2699. 10.1523/JNEUROSCI.0567-09.2010 20164353 PMC2835496

[B162] Vickers S. (1989). Topology via Logic. Cambridge: Cambridge University Press.

[B163] Vreugdenhil M. Toescu E. (2005). Age-dependent reduction of γ oscillations in the mouse hippocampus *in vitro*. Neuroscience 132, 1151–1157. 10.1016/j.neuroscience.2005.01.025 15857717

[B164] White A. Best P. (2000). Effects of ethanol on hippocampal place-cell and interneuron activity. Brain Res. 876, 154–165. 10.1016/S0006-8993(00)02629-9 10973604

[B165] Whittington M. Faulkner H. Doheny H. Traub R. (2000a). Neuronal fast oscillations as a target site for psychoactive drugs. Pharmacol Ther. 86, 171–190. 10.1016/S0163-7258(00)00038-3 10799713

[B166] Whittington M. Traub R. Kopell N. Ermentrout B. Buhl E. (2000b). Inhibition-based rhythms: experimental and mathematical observations on network dynamics. Int. J. Psychophysiol. 38, 315–336. 10.1016/S0167-8760(00)00173-2 11102670

[B167] Wills T. Lever C. Cacucci F. Burgess N. O'Keefe J. (2005). Attractor dynamics in the hippocampal representation of the local environment. Science 308, 873–876. 10.1126/science.1108905 15879220 PMC2680068

[B168] Wilson M. McNaughton B. (1993). Dynamics of the hippocampal ensemble code for space. Science 261, 1055–1058. 10.1126/science.8351520 8351520

[B169] Wixted J. Goldinger S. Squire L. Kuhn J. Papesh M. Smith K.. (2018). Coding of episodic memory in the human hippocampus. Proc. Natl. Acad. Sci. U.S.A. 115, 1093–1098. 10.1073/pnas.1716443115 29339476 PMC5798361

[B170] Wood E. Dudchenko P. Robitsek R. Eichenbaum H. (2000). Hippocampal neurons encode information about different types of memory episodes occurring in the same location. Neuron 27, 623–633. 10.1016/S0896-6273(00)00071-4 11055443

[B171] Wu C. Schulz E. Garvert M. Meder B. Schuck N. (2020). Similarities and differences in spatial and non-spatial cognitive maps. PLoS Comput. Biol. 16:e1008149. 10.1371/journal.pcbi.1008149 32903264 PMC7480875

[B172] Wu X. Foster D. (2014). Hippocampal replay captures the unique topological structure of a novel environment. J. Neurosci. 34, 6459–6469. 10.1523/JNEUROSCI.3414-13.2014 24806672 PMC4012305

[B173] Yartsev M. Ulanovsky N. (2013). Representation of three-dimensional space in the hippocampus of flying bats. Science 340, 367–372. 10.1126/science.1235338 23599496

[B174] Zomorodian A. (2005). Topology for Computing. Cambridge: Cambridge University Press.

[B175] Zomorodian A. Carlsson G. (2005). Computing persistent homology. Discrete Comput. Geom. 33, 249–274. 10.1007/s00454-004-1146-y

